# A penetratin-derived peptide reduces the membrane permeabilization and cell toxicity of α-synuclein oligomers

**DOI:** 10.1016/j.jbc.2022.102688

**Published:** 2022-11-10

**Authors:** Mitra Pirhaghi, Signe Andrea Frank, Parvez Alam, Janni Nielsen, Vita Sereikaite, Arpit Gupta, Kristian Strømgaard, Maria Andreasen, Deepak Sharma, Ali Akbar Saboury, Daniel Erik Otzen

**Affiliations:** 1 Interdisciplinary Nanoscience Centre (iNANO), Aarhus University, Aarhus C, Denmark; 2 Institute of Biochemistry and Biophysics, University of Tehran, Tehran, Iran; 3 Department of Biomedicine, Aarhus University, Aarhus C, Denmark; 4 Department of Drug Design and Pharmacology, University of Copenhagen, Copenhagen Ø, Denmark; 5 Council of Scientific and Industrial Research-Institute of Microbial Technology, Chandigarh, India; 6 G.N. Ramachandran Protein Centre, Academy of Scientific & Innovative Research, Chennai, India

**Keywords:** Parkinson’s disease, peptide array, peptide inhibitors, α-synuclein, oligomer, lipid vesicles, amyloid fibrillation, cell penetrating peptides, Aβ, amyloid-β peptide, αSOs, oligomeric species, α-syn, α-synuclein, ATR, attenuated total reflectance, BBB, blood–brain barrier, CPP, cell-penetrating peptide, DOPG, 1, 2-dioleoyl-*sn*-3-phosphatidylglycerol, LUV, large unilamellar vesicle, MTT, 3-(4, 5-dimethylthiazol-2-yl)-2,5-diphenyltetrazolium bromide, NAC, non–amyloid beta component, PD, Parkinson’s disease, *R*_*h*_, hydrodynamic radius, RT, room temperature, TEM, transmission electron microscopy, ThT, thioflavin T, Trp, tryptophan, Tyr, tyrosine

## Abstract

Parkinson's disease is a neurodegenerative movement disorder associated with the intracellular aggregation of α-synuclein (α-syn). Cytotoxicity is mainly associated with the oligomeric species (αSOs) formed at early stages in α-syn aggregation. Consequently, there is an intense focus on the discovery of novel inhibitors such as peptides to inhibit oligomer formation and toxicity. Here, using peptide arrays, we identified nine peptides with high specificity and affinity for αSOs. Of these, peptides p194, p235, and p249 diverted α-syn aggregation from fibrils to amorphous aggregates with reduced β-structures and increased random coil content. However, they did not reduce αSO cytotoxicity and permeabilization of large anionic unilamellar vesicles. In parallel, we identified a non–self-aggregating peptide (p216), derived from the cell-penetrating peptide penetratin, which showed 12-fold higher binding affinity to αSOs than to α-syn monomers (*K*_*d*_^app^ 2.7 and 31.2 μM, respectively). p216 reduced αSOs-induced large anionic unilamellar vesicle membrane permeability at 10^−1^ to 10^−3^ mg/ml by almost 100%, was not toxic to SH-SY5Y cells, and reduced αSOs cytotoxicity by about 20%. We conclude that p216 is a promising starting point from which to develop peptides targeting toxic αSOs in Parkinson's disease.

Parkinson’s disease (PD) is the second most prevalent age-related neurodegenerative disease, affecting 1 to 2% of the human population older than 60 years (1, 2). Two characteristic features of PD are Lewy bodies and Lewy neurites (3, 4), lead to the progressive loss of dopaminergic neurons in the substantia nigra pars compacta resulting in motor impairment and movement disorders (5). The main component of Lewy bodies is the protein α-synuclein (α-syn), which aggregates to β-sheet–rich amyloid fibrils (6). PD mostly occurs sporadically, but genetically inherited PD accounts for ∼15% of all cases (7). However, the physiological function of α-syn is largely unknown.

α-syn is a 140-residue, 14.5 kDa, highly conserved intrinsically disordered protein found abundantly in presynaptic nerve terminals (4, 6). The primary structure of native α-syn is divided into three domains: the lipid-binding amphipathic N-terminal region (residues 1–60), the hydrophobic self-aggregating non–amyloid beta component or NAC region (61–95) that may contribute to α-syn oligomerization and aggregation, and the acidic unstructured C-terminal tail (96–140) (8, 9, 10).

Although the normal physiological functions of α-syn remain poorly understood, suggestions include a role in neurotransmitter release (11, 12), synaptic plasticity (13), learning (14), maintenance of SNARE protein complexes (11), maintenance of synaptic vesicle pools (15, 16, 17), protein-folding chaperone (18), dopamine neurotransmission (17), and endoplasmic reticulum–Golgi trafficking (19). It is generally accepted that α-syn interactions with membrane play a role in the physiological and pathological function of the protein (20, 21, 22).

Both α-syn monomers and oligomers (αSO) show particular affinity for negatively charged lipid vesicles (23, 24, 25). Under fibrillating conditions, α-syn forms different soluble oligomers (on- and off-pathway) (26, 27, 28), which are the most important toxic species to cells in culture (more toxic by weight than fibrils) and are able to disrupt membranes, leading to mitochondrial dysfunction, oxidative stress, and finally cell death (25, 29, 30, 31, 32, 33, 34).

Polyphenolic compounds (35, 36, 37, 38, 39) have been studied as amyloid inhibitors for many years; however, their effects are very general and unspecific, whereas protein–protein interactions tend to be very specific and well regulated. Peptides are promising candidates for drug development because of their small size, functional diversity, high biological activity, low production costs, high membrane penetration ability, and high target affinity (40, 41). α-syn self-recognition makes peptides even more attractive, for example, peptides corresponding to the NAC region would be expected to bind to the NAC region and thus inhibit amyloid fibril formation. Indeed, over the past 2 decades, numerous peptidic inhibitors have been designed to prevent α-syn fibrillation and oligomerization. These include modified short synthetic peptides, derived from amyloidogenic sequences of α-syn (residues 64–100) (42, 43), N-methyl peptides, derived from residues 77 to 82 and 68 to 78 of α-syn (44, 45, 46), pyrroloquinoline quinone-modified α-syn 36 to 46 peptide (47), first 15 N-terminal residues of the nonaggregative α-syn homolog β-synuclein (48, 49, 50), screened peptides from 10^6^ random amino acid sequences (51), and biomimetic peptides designed using a new database (ProDa) (52). While modified peptides derived from amyloidogenic sequences of α-syn are fibrillation inhibitors, unmodified homologous peptides can accelerate fibrillation and oligomerization (53). On the other hand, amyloid-β peptides (Aβ 1–42 and pGlu-Aβ 3–42) also accelerate α-syn fibril formation (54). Cyclic peptides and their derivatives also show promise as inhibitors of fibrillation and cytotoxicity of aggregates (55, 56).

Cell-penetrating peptides (CPPs) (57) are defined as a short relatively nontoxic and partly hydrophobic and/or polybasic peptides (at most 5–35 amino acid residues), with a net positive charge at physiological pH. CPPs have the ability of interacting with negatively charged groups on the cellular membrane, which results in their internalization (58, 59) but can also cotransport a variety of cargoes inside the cells (58, 60, 61, 62). Cationic CPPs are able to translocate across membranes in a nonendocytotic fashion, generally by one of following mechanisms: the inverted micelle model, carpet model, and pore formation model. CPPs could cause cytoplasmic leakage and toxicity in different cell lines because of membrane disruption and also interference with the functioning of membrane proteins (62), endowing them with antiviral, antitumoral, and antimicrobial activity (57). One of the most widely used CPPs is pAntp (43, 44, 45, 46, 47, 48, 49, 50, 51, 52, 53, 54, 55, 56, 57, 58) (also known as penetratin, sequence RQIKIWFQNRRMKWKK), which derives from the third helix of the Antennapedia protein homeodomain (57, 63). Penetratin has been shown to inhibit fibrillation of α-syn (64), making it a promising starting point for peptides able to bind specifically to αSOs.

Here, we used peptide arrays to identify peptides targeting αSOs. About 384 different peptides were displayed on the array, representing a range of potential binding motifs. Peptides 1 to 127 are 14-mers spanning the 140-residue sequence of α-syn, whereas peptides 128 to 149 and 151 to 183 are peptides identified from a phage display analysis of peptides binding to immobilized monomeric α-syn. Peptide 184 is the CPP penetratin, whereas peptide 185 (sequence KLAKLAKKLAKLAK, abbreviated KLA) is a proapoptotic peptide that induces apoptosis in the cell (65). Peptides 186 to 254 and 255 to 279 are modified versions of penetratin and KLA respectively, made by Ala-scanning, N- and C-terminal truncation, hydrophobic modifications, and scrambled versions of the peptides (sequences provided in Table S1). To evaluate the relative binding efficacies of these peptides, we performed competition experiments in which a constant concentration of fluorescently labeled αSOs was challenged with different concentrations of unlabeled monomeric α-syn, and the extent of αSO binding was then determined by scanning the peptide array. Fluorescence intensities of each spot were plotted against [α-syn] and fitted as a reversed binding isotherm to obtain apparent dissociation constants (*K*_*d*_^app^). We ranked peptides based on the fitting data and selected the top nine peptides (p5, p12, p162, p168, p216, p194, p222, p235, and p249), which were subsequently synthesized. We then investigated the impact of these peptides on α-syn fibrillation as well as on the ability of αSO to permeabilize large unilamellar vesicles (LUVs) formed by 1, 2-dioleoyl-*sn*-3-phosphatidylglycerol (DOPG). We also analyzed the peptide’s toxicity toward SH-SY5Y cells and their ability to rescue cells against αSO toxicity. We find that one peptide (p216) is particularly promising, being able to reduce cell toxicity and membrane permeability of αSOs.

## Results

### Peptides showing higher affinity for αSOs rather than monomers

**Figure 1:**
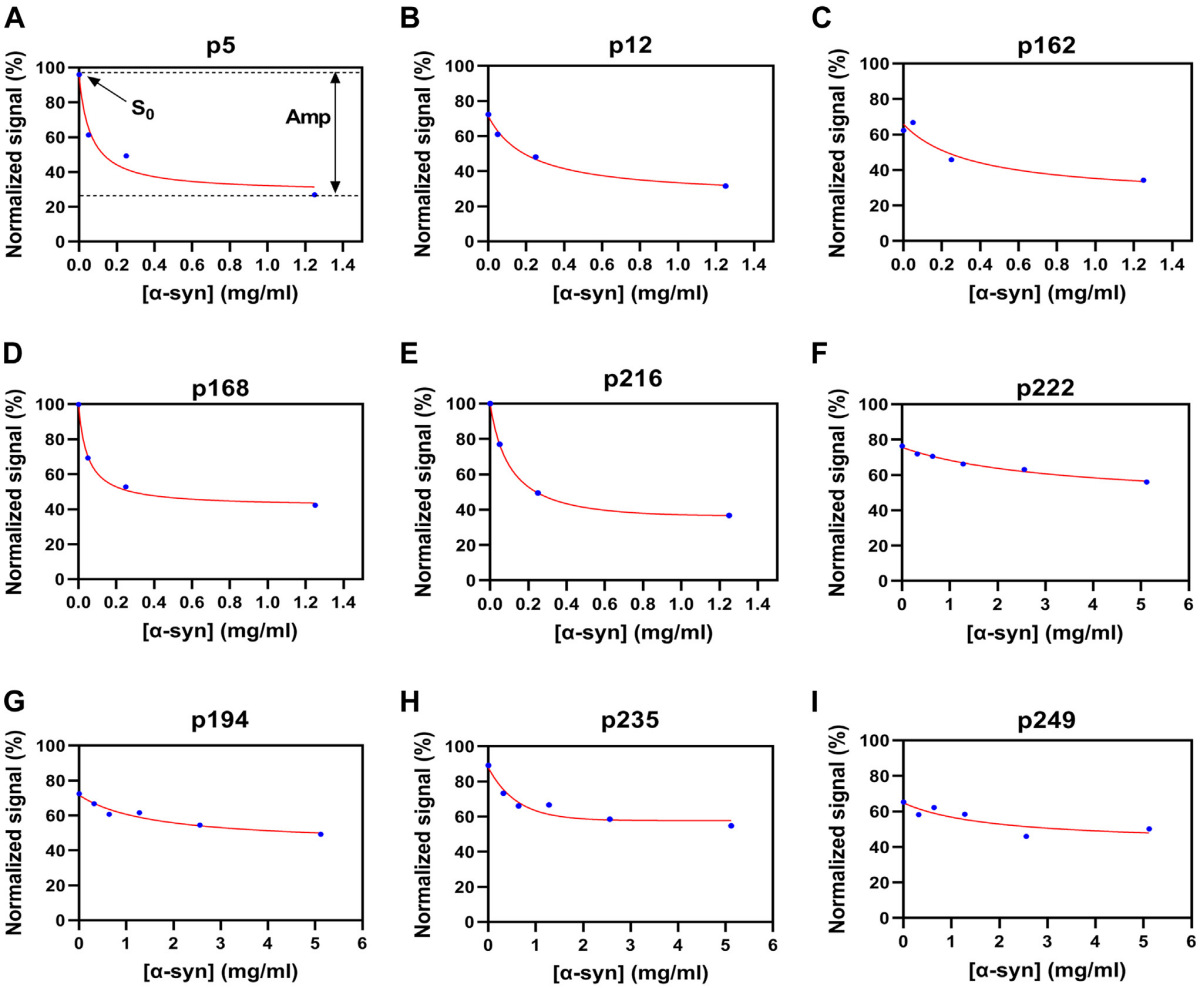
**Normalized fluorescence intensity on the array *versus* α-syn concentration for the nine best peptides.** All 9 curves (A-I) were fitted as a reversed binding isotherm (Equation 4). α-syn, α-synuclein.

CelluSpots peptide arrays were used to identify peptides with higher affinity for αSOs than for monomeric α-syn. To accomplish this, we carried out competition experiments (Fig. S1) in which AlexaFluor 546-labeled αSOs and unlabeled α-syn monomers were added in different mass ratios (1:0, 1:1, 1:5, and 1:25 for the first experiment and 1:0, 1:1, 1:2, 1:4, 1:8, and 1:16 for the second experiment). The extent of decrease of fluorescence intensity reports on how well monomeric α-syn outcompetes αSOs for binding to the peptides. Fluorescence signals (normalized against the highest value on the array) were plotted against different α-syn concentrations (Fig. 1) and fitted as a reversed binding isotherm (Equation 4) (66) to obtain apparent dissociation constants (*K*_*d*_). We ranked peptides based on the fitting data (Equation 5), prioritizing peptides with high starting fluorescence signals S_0_ (indicating high levels of binding of the oligomer), high *K*_*d*_ values (low affinity for the monomer), and low amplitudes (low levels of αSO displacement from the displayed peptide).

(4) $$Y=-\text{Amp}\times \left(\frac{X}{X+K_{d}}\right)+S_{0}$$

(5) $$\text{weighted}\;\text{value}=S_{0}-\text{Amp}+{f\;K}_{d}$$

Here, *S*_*0*_ is starting intensity, *Amp* is the amplitude of signal change, *K*_*d*_ is the apparent dissociation constant, and *f* is the correction factor.

Binding data for the nine best peptides are shown in Figure 1 and summarized in Table 1. Additional peptide data are shown in Fig. S2.

**Table 1:** Properties of the nine best peptides from two competition peptide array experiments

Array	Peptide	Sequence	Origin	Molecular weight (Da)	Net charge (pH 7)
First	p5	MKGLSKAKEGVVAA	α-syn sequence 5–18	1388.69	+2
First	p12	KEGVVAAAEKTKQG	α-syn sequence 12–25	1415.61	+1
First	p162	SASDSRTFKSSGG	Phage display peptides	1286.32	+1
First	p168	SASSHLHHGHSGG	Phage display peptides	1270.29	+0.9
Both	p216	WFQNRRMKWKK	N-terminal truncation of penetratin	1607.94	+5
Second	p222	RQIKIWFQNRRFKWKK	Modulating hydrophobicity of penetratin^a^	2203.73	+7
Second	p194	RQIKIWFQARRMKWKK	Ala scan of penetratin^a^	2262.74	+7
Second	p235	NQKIKWRWIQRFRKKM	Scrambled version of penetratin^a^	2246.75	+7
Second	p249	MKQINRWFIRWQKKRK	Scrambled version of penetratin^a^	2246.75	+7

a Penetratin has the sequence RQIKIWFQNRRMKWKK.

### Effect of selected peptides on α-syn fibrillation and secondary structure

**Figure 2:**
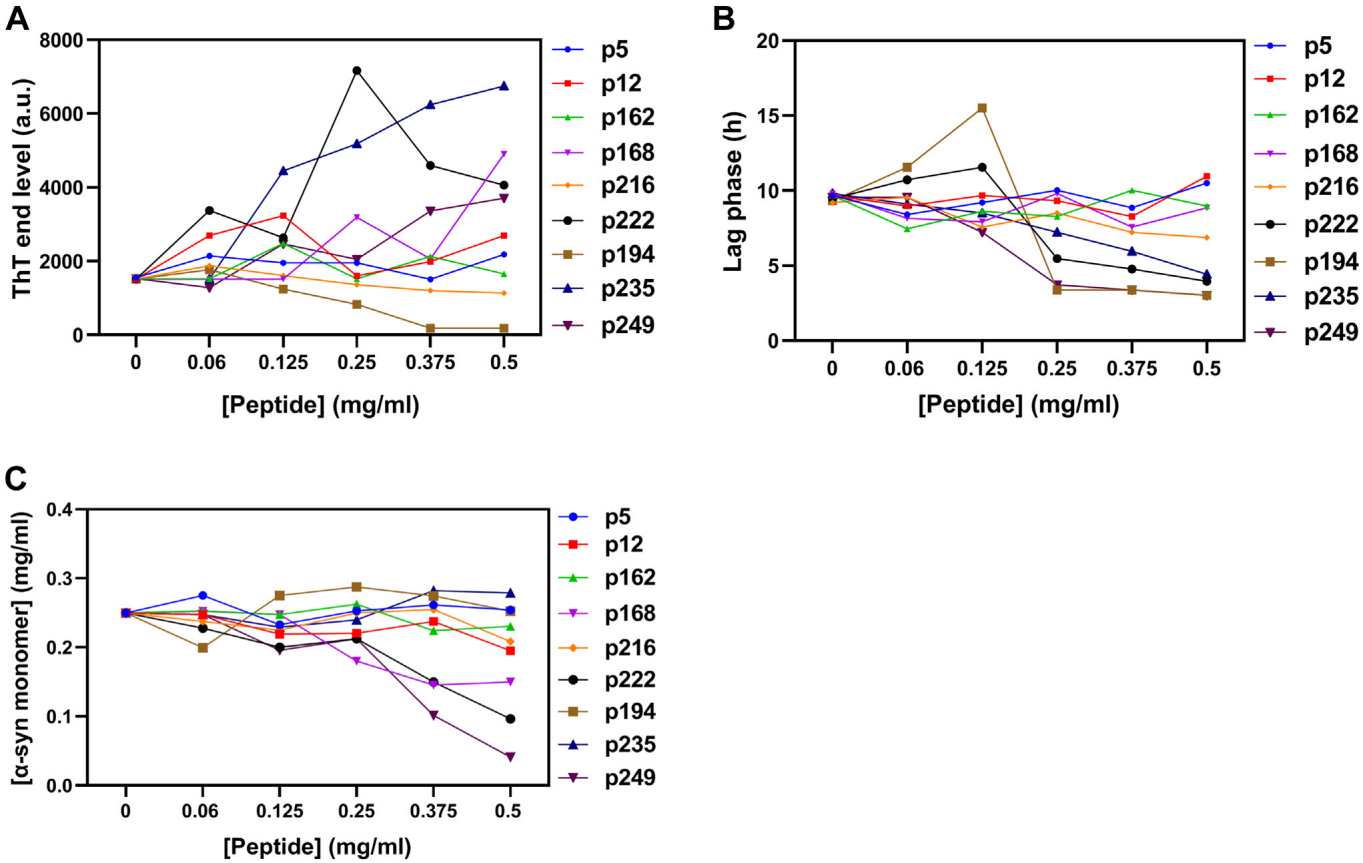
**Effect of peptides on α-syn fibrillation.** Plots of the lag phase time (*A*) and ThT signal end level (*B*) of unseeded aggregation kinetics *versus* the peptides concentration. *C*, quantification of the SDS-PAGE results for supernatants of all samples after the end of incubation. The intensity of all bands at 14.4 kDa (α-syn) was determined using ImageJ, and the α-syn monomer concentrations left in supernatant were achieved using α-syn standard band (are not shown) before fibrillation. Raw data are shown in Fig. S3. α-syn, α-synuclein; ThT, thioflavin T.

To test the effects of these selected nine peptides on the aggregation of α-syn, we performed a thioflavin T (ThT) fluorescence assay in the presence of 1 mg/ml α-syn and 0 to 0.5 mg/ml peptide for 48 h (raw data in Fig. S3). Furthermore, the peptides alone were monitored for self-fibrillation. Only p222 showed slight self-fibrillation tendencies. Five peptides (p5, p12, p162, p168, and p216) did not change the lag phase duration (Fig. 2*A*), rate of aggregation, and ThT signal end level (Fig. 2*B*). In contrast, p194 showed an inhibitory effect, whereas p222, p235, and p249 had a slight aggregation-promoting effect at high concentrations with a shorter lag phase (Figs. S3, *F*–*I* and2, *A* and *B*). High concentrations of p222, p194, and p249 led to a decline in ThT fluorescence after reaching a plateau, possibly because of the formation of insoluble amorphous species. We also carried out seeding experiments by adding 5% sonicated preaggregated α-syn fibrils to the ThT assay. However, no peptides changed the lag phase and rate of the growth phase (Fig. S4), indicating that they do not interfere with elongation or secondary fibrillation processes. Attempts to analyze the various ThT time profiles by the webserver AmyloFit (67) to obtain more detailed mechanistic insights did not yield robust conclusions, in part because of the irregular and nonsigmoidal shape of many of the fibrillation profiles and in part because of the relatively small variation in kinetics for the profiles that could be analyzed.

SDS-PAGE was used to analyze the amount of soluble α-syn in the supernatant after centrifugation. p5, p12, p162, and p216 do not affect the amount of monomer left in the supernatant, whereas p168, p222, and p249 decrease solubility (Figs. 2*C* and S5). Overall, there was a reasonable but not perfect correspondence between SDS-PAGE and ThT data.

**Figure 3:**
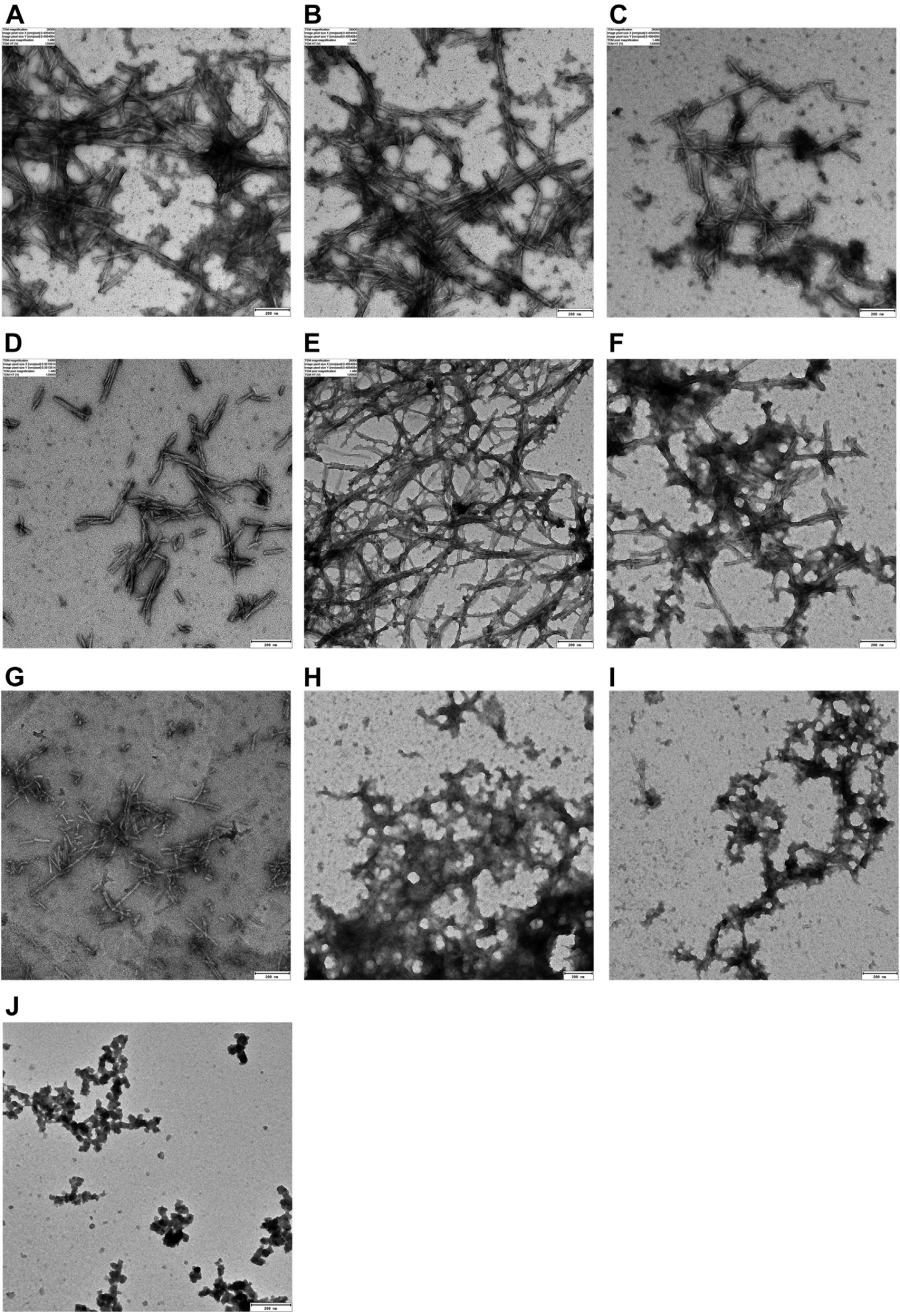
**TEM image of α-syn aggregates after incubation with peptides.** α-syn incubated alone (*A*) and in the presence of peptides (*B*–*J*). The scale bars represent 200 nm. α-syn, α-synuclein; TEM, transmission electron microscopy.

The morphology of the α-syn aggregates formed in the presence of peptides was investigated by transmission electron microscopy (TEM) microscopy (Fig. 3*A*). α-syn forms long twisted fibrils, and p5, p12, p162, and p216 (Fig. 3, *B*–*D*, and *F*) did not cause any significant change in morphology (shape, length, and thickness) of fibrils. These data broadly support the previous results. However, in the presence of p168 (Fig. 3*E*), α-syn produced longer and thinner fibrils, and the number of fibrils increased. p194, p235, and p249 (Fig. 3, *H*–*J*) also caused nonfibrillar (amorphous) accumulations (the decline of ThT fluorescence). However, α-syn formed needle-shaped and short fibrils in the presence of p222.

**Table 2:** ATR–FTIR secondary structural changes of samples at the end of incubation in the presence of different concentrations of peptides

Samples	Secondary structural changes^a^		
	Coil (1662–1686 cm^−1^)	Random (1642–1660 cm^−1^)	Cross β-sheet (1610–1630 cm^−1^)
α-syn + p5	↑	↑	↓
α-syn + p12	↑	↑	↓
α-syn + p162	≈	≈	≈
α-syn + p168	≈	≈	≈
α-syn + p216	≈	≈	≈
α-syn + p222	↑↑	↑↑	↓↓
α-syn + p194	↑↑↑	↑↑↑	↓↓↓
α-syn + p235	↑↑↑	↑↑↑	↓↓↓
α-syn + p249	↑↑↑	↑↑↑	↓↓↓

a ↑, ↓, and ≈ represent an increase, decrease, and no change of the corresponding secondary structure with increasing concentration of peptides, respectively. The number of arrows indicates the extent of change.

**Table 3:** Comparison of ThT, SDS-PAGE gel, far-UV CD, ATR–FTIR, and TEM data on each peptide^a^

Peptides	p5	p12	p162	p168	p216	p194	p222	p235	p249
Lag phase time duration	≈	≈	≈	≈	≈	↑^b^	↓	↓	↓
ThT signal end level	≈	≈	≈	≈	≈	↓↓	↑↑	↑↑	↑
Soluble α-syn left in supernatant (SDS-PAGE gel)	≈	≈	≈	↓	≈	↑	↓↓	≈	↓↓
ATR–FTIR	↓ amyl	↓ amyl	≈	≈	≈	↓↓↓ amyl	↓↓ amyl	↓↓↓ amyl	↓↓↓ amyl
Far-UV CD	↓β	↓β	↓β	↓↓β	↓↓β	↓↓↓β	↓↓↓β	↓↓↓β	↓↓↓β
Morphology (TEM)	≈	≈	≈	L, T, I	≈	A	S, N	A	A

a A, amorphous aggregates; amyl, amyloid structures; β, β-sheet structures; L, S, T, N, and I, long, short, thin, needle-shaped, and increased fibril number, respectively. ↑, ↓, and ≈ represent an increase, decrease, and no change (compared with control, α-syn alone), respectively. The number of arrows indicates the extent of change.

b Signal decline at high concentrations.

**Figure 4:**
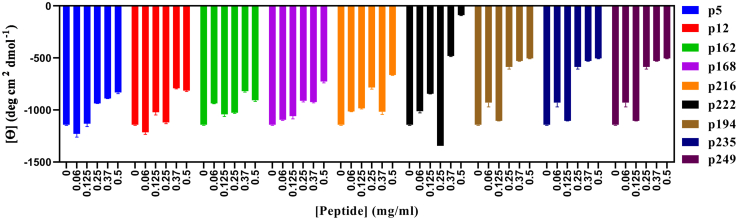
**Changes in molar ellipticity at 218 nm after incubation of 1 mg/ml α-syn in the presence of different concentrations of peptides for 48 h.** α-syn, α-synuclein.

To analyze the secondary structure of the final aggregates, we recorded far-UV CD and attenuated total reflectance (ATR)–FTIR spectra for fibrillated samples after washing to remove free α-syn and peptides. According to far-UV CD (Fig. S6, *A*–*I*), all α-syn samples had significant β-sheet structures with a minimum around 218 nm, with slightly increased intensity and minor red shifts at some points. Also all peptides alone before incubation showed dominant random coil structure (Fig. S6*J*). Overall, as the concentration of peptides increases, the intensity of signal at 218 nm decreases (Fig. 4*A*). We observed that α-syn fibrils formed in the presence of p194, p222, p235, and p249 precipitated rapidly after mixing with these peptides. This likely gives rise to light scattering, which reduces CD signals. To overcome these artifacts, we turned to ATR–FTIR spectroscopy, which is not sensitive to scattering. For amyloid fibrils, ATR–FTIR shows a distinct amide I band that extends from 1611 cm^−1^ to 1630 cm^−1^ (Fig. S7) (68). Both far-UV CD and ATR–FTIR spectroscopy confirm extensive β-sheet structure. However, in the presence of peptides, some structural changes were observed as reflected by the decrease in the amyloid cross β-sheet structure at ∼1629 cm^−1^ and increases in the random and coil structures (∼1656 and ∼1676 cm^−1^, respectively) (Table 2). The amount of amyloid structures decreased in the presence of p222, p194, p235, and p249, especially at high concentrations. p5 and p162 slightly decreased the amount of amyloid. On the other hand, p12, p168, and p216 did not show significant changes in FTIR spectra. Taking all together, ATR–FTIR data are in reasonable agreement with far-UV CD. For ease of comparison, data from all tests after fibrillation in the presence of peptides are summarized in Table 3. FTIR spectra for peptides alone before incubation are shown in Fig. S8 and summarized in Table S2.

### Effect of αSOs–peptides on membrane permeabilization and cell viability

**Figure 5:**
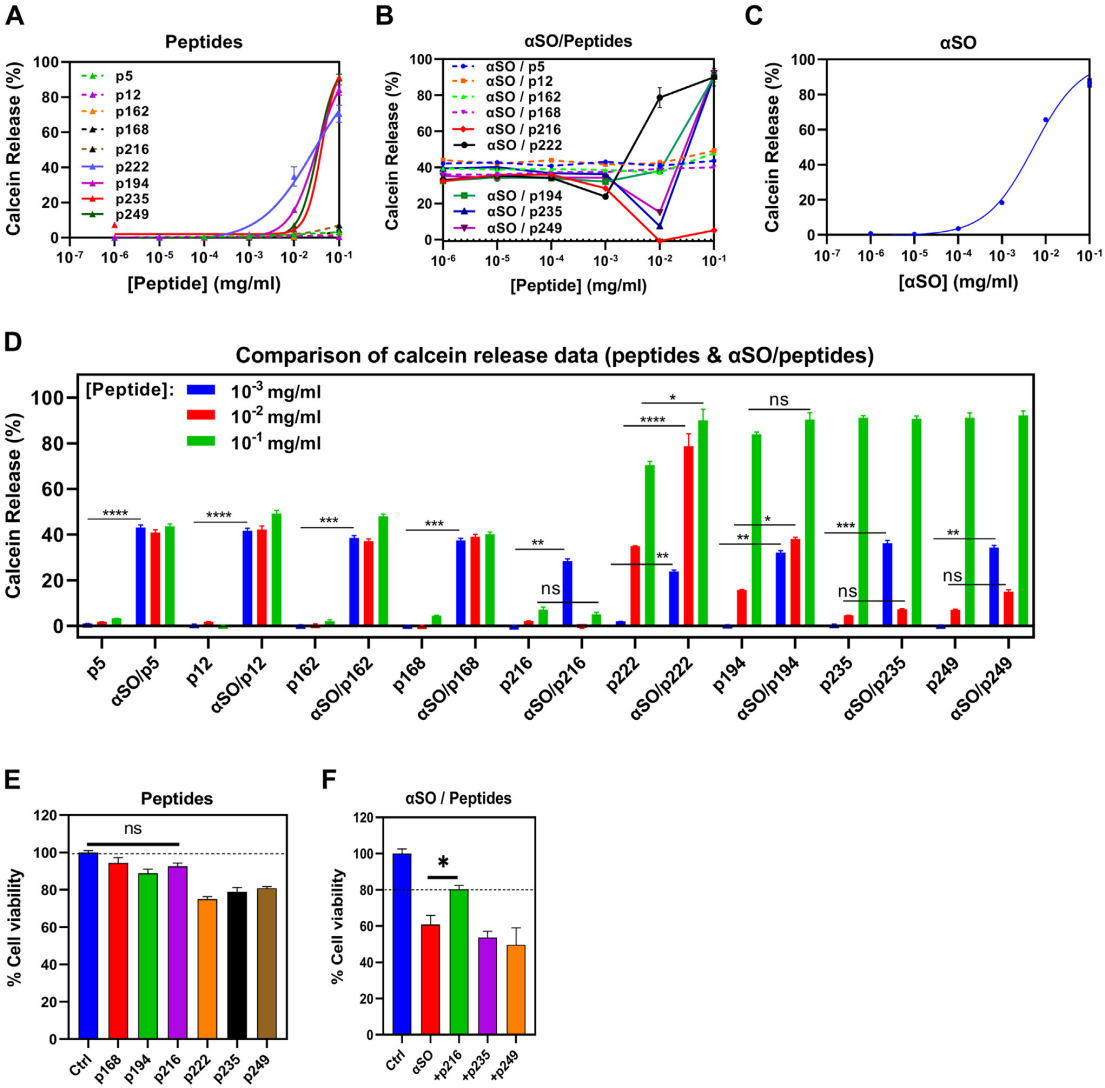
**Effect of peptides on α-syn oligomer membrane permeabilization and cell toxicity.***A*–*C*, calcein release assay from calcein-loaded DOPG vesicles in the presence of (*A*) αSOs, (*B*) peptides, (*C*) αSOs–peptides. *D*, comparison of calcein release data (peptides and αSOs–peptides) (two-way ANOVA, ∗*p* < 0.03, ∗∗*p* < 0.002, ns). *E* and *F*, MTT cell viability assays with SH-SY5Y cells (*E*) using 35 μg/ml peptides alone and (*F*) 0.14 μg/ml αSOs together with selected peptides (∗*p* < 0.05, ns). αSOs, oligomeric species; DOPG, 1, 2-dioleoyl-*sn*-3-phosphatidylglycerol; MTT, 3-(4, 5-dimethylthiazol-2-yl)-2,5-diphenyltetrazolium bromide; ns, not significant.

αSOs show cytotoxic properties, which have been attributed to their membrane-disrupting ability (69). To examine the effect of peptides on membrane permeabilization by αSOs, we used a simple calcein release assay. αSOs, peptides, and aSO–peptide mixtures at 10^−6^ to 10^−1^ mg/ml peptide were added to calcein-loaded DOPG vesicle, and the extent of calcein release was monitored for 30 min by the increase in fluorescence as calcein leaks out upon membrane permeabilization, leading to dilution and the loss of self-quenching. The time-dependent calcein release data from DOPG vesicles (70, 71) in the presence of peptides, αSOs, and αSO–peptides are shown in Figs. S9 and S10. In order to allow easy comparison of all peptides, the calcein release *versus* concentration (peptides and αSOs) were plotted at the plateau point of release (Fig. 5, *A*–*C*). As summarized in Figure 5*A*, p5, p12, p162, p168, and p216 alone did not permeabilize lipid vesicles. However, peptides 194, 222, 235, and 249 led to vesicle permeabilization, giving rise to 50% calcein release from vesicles (calcein^50%^) at 0.03 to 0.04 mg/ml. αSOs alone showed 50% calcein release at 5.2 μg/ml (Fig. 5*C*). Incubation of αSOs at its calcein^50%^ with increasing peptide concentration (Fig. 5*B*) led to increased vesicle permeabilization by αSOs for p222 at 0.01 and 0.1 mg/ml and for p194, p235, and p249 only at 0.1 mg/ml. p5, p12, p162, and p168 failed to affect vesicle permeabilization by αSOs. On the other hand, p235 and p249 at 0.1 mg/ml and p216 at 0.01 and 0.1 mg/ml led to inhibition of membrane permeabilization by αSOs. Remarkably, 0.1 mg/ml of p216 led to complete inhibition of permeabilization. Figure 5*D* compares calcein release percentages of data represented in Figure 5, *A* and *B* at peptide concentrations between 0.001 and 0.1 mg/ml, where we see the largest extent of calcein release in general.

The effect of the peptides on αSOs toxicity in SH-SY5Y cells using 3-(4, 5-dimethylthiazol-2-yl)-2,5-diphenyltetrazolium bromide (MTT) cell viability assay was also tested to complement our calcein data. We selected three effective peptides (p216, p235, and p249), two toxic peptides (p194 and p222), and one ineffective peptide (p168). We used 10 μM αSOs (0.14 μg/ml), which reduces the viability up to 60 to 70% and combined this with low levels of peptide (35 μg/ml). By themselves, some of the peptides showed cell toxicity at these concentrations (Fig. 5*E*). As predicted by calcein release data, p222 was more toxic than the other peptides (74% cell viability) and p168 was less toxic (94% cell viability). Two of the effective peptides (p235 and p249) slightly reduced cell viability to 78 and 80%, respectively. Gratifyingly, p216 only reduced 92% cell viability to 92% at this concentration. When αSO is included, the oligomer alone decreased viability by 40% compared with control (untreated cells). Among the different peptides tested together with αSO, only p216 was able to counteract this, increasing viability by ca. 20% compared with αSOs alone (Fig. 5*F*).

### Binding affinity of the selected best peptide to αSOs and α-syn

We turned to tryptophan (Trp) anisotropy to determine the binding of effective peptides to αSOs, since p194, p222, p216, p235, and p249 all have Trp residues, whereas αSOs has none. Thus, Trp anisotropy should report on the reduction in mobility of the peptides caused by interaction with αSOs. All peptides show an increase in anisotropy when incubated with αSOs (Fig. S11), indicating an interaction with the oligomer in all cases. However, it was difficult to obtain more specific information about the affinity.

**Figure 6:**
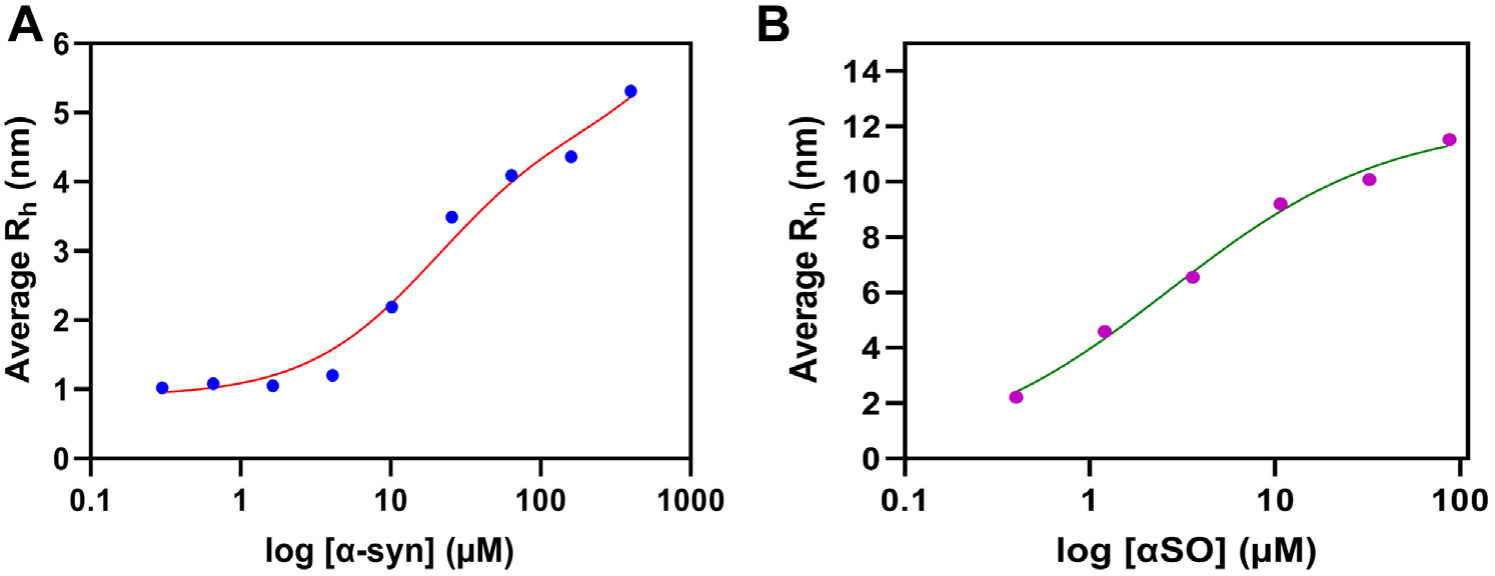
**Plots of the hydrodynamic radius (*R***_***h***_**) of p216 *versus* log[α-syn] or log[αSOs].** Data are fitted with a 1:1 binding isotherm, yielding an apparent dissociation binding constant (*K*_*d*_) of 31.2 and 2.7 μM for α-syn and αSO, respectively. αSOs, oligomeric species; α-syn, α-synuclein.

To determine an actual binding affinity of p216 to αSOs and α-syn, the hydrodynamic radius (*R*_*h*_) of labeled p216 bound to unlabeled αSOs–α-syn was determined by microfluidic diffusional sizing (72). An advantage of microfluidics studies is that they require very little material (unlike, *e.g.*, isothermal titration calorimetry) and do not require immobilization of flexible molecules (as is required for, *e.g.*, surface plasmon resonance), which can otherwise lead to artifacts. Rather, a direct and easily interpretable parameter is provided in the form of the *R*_*h*_. As shown in Figure 6, monomeric α-syn has a much lower affinity for p216 than αSO does. The data could be fitted with a 1:1 binding scheme, giving a dissociation binding constant (*K*_*d*_) of 31.2 and 2.7 μM for α-syn and αSO, respectively. For both species, binding of peptide led to a significant increase in *R*_*h*_, confirming peptide immobilization in complex with these states. The measured values of ca. 5 and 11 nm for α-syn and αSO, respectively, agree nicely with the known *R*_*h*_ of α-syn (73) (4 nm) and αSO (10 nm) (74).

## Discussion

### Peptides with higher affinity to αSO than α-syn have different effects on α-syn fibrillation

This study focuses on peptides identified from peptide arrays to have high affinity toward αSOs. Our competition data show that they also bind to monomeric α-syn but to a smaller extent. Arguably, the ideal peptide inhibits α-syn fibrillation as well as reducing αSOs toxicity. Nevertheless, our biophysical analyses reveal that peptides p5, p12, p162, p168, and p216 had very little effect on the fibrillation process as measured by the lag phase duration, ThT end level, and the amount of soluble α-syn remaining in solution; furthermore, they had little if any effect on the secondary structure or overall morphology of the ensuing α-syn fibrils. These results are not surprising given that the peptides were selected based on their high binding affinity to the αSOs.

p235 and p249 deviate modestly from this overall pattern. These peptides slightly reduced the lag phase duration and the amount of soluble α-syn remaining in the solution but increased the ThT end level, indicating an appearance accelerating effect on the α-syn fibrillation process. However, they only slightly reduced ThT end levels in seeding experiments (even at high peptide concentrations) without any changes in the elongation rate and secondary nucleation process. TEM images, the visual appearance of white deposits in the solution at the end of incubation (especially at high peptide concentrations), and the reduction in β-sheet structures suggest that α-syn has formed amorphous aggregates. Such large insoluble aggregates will lead to light scattering and could explain the irregular ThT fluorescence time course.

Unlike other peptides, p222 forms aggregates by itself with a very modest increase in ThT signal after a lag phase of 3 to 4 h. The FTIR spectrum of p222 confirms the presence of amyloid. However, the ThT kinetics of p222 aggregation are not affected by α-syn seeds, indicating a lack of crosstalk between α-syn monomers and p222 aggregates. The peptide p194 reduces the lag phase and end point of the ThT time profile, slightly increases the amount of α-syn monomers left in solution, and–like p235 and p249–causes α-syn amorphous accumulation (Fig. 3*H*).

### The penetratin-derived peptide p216 reduces αSO-induced membrane permeability and cytotoxicity, making it a promising candidate for therapy

Next, using calcein-filled DOPG LUVs, we investigated the effect of these peptides on membrane permeabilization by αSOs. p5, p12, p162, p168, and p216 alone, in the tested concentration range (10^−1^–10^−6^ mg/ml), did not affect vesicle permeability. However, p222 shows membrane toxicity from >10^−4^ mg/ml, whereas p194, p235, and p249 show toxicity >10^−2^ mg/ml. p222, p194, p235, and p249, as well as p216, are derivatives of penetratin. Penetratin forms β-structures on vesicles formed by DOPG and other negatively charged lipids (at concentrations >10^−3^ mg/ml), leading to membrane perturbation and permeabilization (75, 76, 77, 78). Among the peptides tested here, p222 is more potent in vesicle permeabilization than p194, p235, and p249, possibly because of the formation of αSOs with increased toxicity against the membrane. Gratifyingly, p216, though a derivative of penetratin, in this concentration range did not affect membrane permeability. On the other hand, p222 increased αSO-induced membrane permeability, probably because of the synergistic effect of β-structures of peptide 222 with αSOs. However, p235, p249, and especially p216 reduced this by 80%, 62%, and 100%, respectively. The vesicle-protective properties of p216 were translated to a favorable performance in cell toxicity assays, whereas slight toxicity was observed by p222, p235, and p249. In the presence of toxic αSOs, p216 increased cell survival by about 20%, whereas p235 and p249 did not show positive effects. Thus, p216 continues to be a leading candidate.

What could be the reason for the superior performance of p216? αSOs binds through electrostatic and hydrophobic interactions to the negatively charged membrane. The positive N-terminal domain of αSOs acts like an anchor, facilitating αSO binding to the bilayer. Subsequently, the hydrophobic core of αSOs (residues 70–88) located within the NAC region leads to the insertion of αSOs structured β-sheet–rich core deeply into the membrane interior (79). p216 has an amphipathic sequence with hydrophobic N terminal and basic C terminal, which may incline it to membrane binding but with a superficial location, which does not disrupt the membrane organization. The membrane-active peptides p235 and p249 are not amphipathic but could conceivably form pores within the membrane instead of binding at the interface (80), leading to membrane toxicity. Perhaps p216 binds to the negatively charged regions of αSOs, neutralizing their charge and thus decreasing their ability to interact with membranes. Similar effects have been reported for the amphipathic peptide PSM3α, which forms a very stable complex with αSOs (81).

Penetratin is a widely used CPP. Translocation of full-length and N-terminal truncated (82) penetratin has been observed in all cell types, and it is also able to cross the blood–brain barrier (BBB) (83). Its mechanism of translocation in live cells is complex and involves structural changes from random coil to α-helix (84) and transient formation of tubes or inverted micelles in the membrane that entrap the peptide in its interior. Translocation has been observed in all cell types. The presence of Trp and net positively charged amino acid residues are important for formation of inverted micelles and peptide import into cells (76, 77, 78, 85). But still, the mechanism of penetration’s uptake in cells remains an open question. At concentrations <10 μM, toxicity is rare and depends on the cell type. On the other hand, partial amphipathicity of penetratin is required for membrane interaction and internalization (76, 85). This study is the first report of how penetratin and its derivatives affect αSO toxicity. A previous study (86) reported the effect of penetratin-derived peptides in inhibiting Aβ fibrillation and provided a general formula for inhibitory peptides (X_1_ [Lys X_2_ X_3_ Phe Gln]_m_ Arg Gln Ile [Lys X_4_ X_5_ Phe Gln]_n_ (X_1_ is H or an acetyl group, X_2_ and X_4_ are Ile/Leu, X_3_ and X_5_ are Pro/Trp, *m* is 0 or 1, *n* is 1 and 2)). These inhibitory peptides inhibit amyloid aggregate formation. pAntp (RQIKIWFQNRRMLWLX_aa_) and pAntp-BSB1 (RQILIWFQNRRMLWLL) are particularly effective inhibitors. These sequences bear some resemblance to our most effective αSO-binding peptide p216 (sequence WFQNRRMKWKK) but are even closer related to p222, p235, and p249, which however were shown to be toxic to cells in our study.

Treatment and prevention of amyloidosis may involve developing agents that hinder amyloid aggregation. Promising examples include short peptides with some sequence homology to the natural protein sequence believed to be involved in amyloid formation but also containing one or more amino acids that disfavor or destabilize the formation of β-pleated sheet conformations (42, 43, 53, 87). Others have developed scrambled peptides derived from the NAC domain of α-syn (88, 89), peptides that have homologous sequences with proteins but are chemically modified (46), or completely new designed peptides by *in silico* panning (90). To date, there are no peptidic inhibitors that specifically target toxic αSOs. As mentioned before, cationic penetration can cross the BBB, and its derivate p216 seem to be better at crossing the cell membrane and by inference also the BBB.

In summary, the penetratin derivative p216 shows amphipathicity, contains Trp and positively charged amino acid residues for cell membrane interaction and internalization, and can likely cross the BBB. Given its ability to target toxic αSOs with high binding affinity and reduce oligomer cytotoxicity, this cationic peptide may serve as a promising compound for the development of therapeutics for PD.

## Experimental procedures

### Materials

DOPG sodium salt was from Avanti Polar Lipids. All other chemicals were of analytical grade from Merck or Sigma–Aldrich.

### Monomeric α-syn production and handling

Recombinant human α-syn was expressed in *Escherichia coli* BL21 (DE3) with a plasmid vector pET11-D containing the α-syn gene (SNCA) and purified using autoinduction accordingly (91, 92) with an additional acid precipitation step (39, 93). Lyophilized α-syn was stored at −20 °C until further use. Before all experiments, lyophilized α-syn was dissolved in PBS buffer (20 mM phosphate, 150 mM NaCl, pH 7.4), and the stock solution was filtered through a 0.22 μm filter (Q-Max RR Syringe Filters; Frisenette ApS). The α-syn concentration was determined using ε_280_ = 0.412 (mg/ml)^−1^ cm^−1^ (94).

### Oligomeric α-syn (αSO) purification

αSOs were prepared as described before (38, 39). Briefly, 553 μM monomeric α-syn in PBS buffer (pH 7.4) was incubated for 4 to 5 h at 37 °C and 900 rpm shaking on an Eppendorf thermoshaker, TS-100 (BioSan). The sample was centrifuged for 10 min at 13,000 rpm, at room temperature (RT) to remove insoluble material, and the supernatant was loaded on a 24 ml Superose 6 10/300 GL SEC column (GE Healthcare Life Sciences) at 2.5 ml/min in PBS buffer. αSO fractions were collected and upconcentrated using Amicon Ultra 0.5 ml Centrifugal Filters (Merck) with a 100 kDa cutoff and stored at −80 °C.

### CelluSpots peptide array

CelluSpots peptide arrays were printed as described (95, 96). Each array contained 384 peptide spots on a cellulose matrix. Spots 1 to 127 displayed 14-mers of the α-syn sequence advancing stepwise one residue. Spots 128 to 185 were peptides identified by phage display screening with α-syn (97). The rest of the peptide array displays different modifications of two other peptides selected on the basis of their cell-penetration abilities. Modifications include alanine scanning, terminal truncations, hydrophobicity changes, and scrambled sequences. The best peptide is a CPP, and the second-best peptide is a proapoptotic peptide (97), which are screened as inhibitors of fibrillation (98). An overview of the array sequences is provided in Table S1. Before each experiment, peptide arrays were blocked in 25 ml 3% skim milk solution (0.75 g skim milk powder in 25 ml MilliQ water) in a Nunc tube on a roller overnight at 4 °C. After blocking, the arrays were transferred to 4-well dishes (nontreated Thermo Scientific Nunc rectangular dishes) and washed in a block buffer (20 mM Tris, pH 7.5, 0.1% Tween-20). AlexaFluor 546-labeled αSOs (A546; Thermo Fisher) in the ratio of αSOs to monomer 1:0, 1:1, 1:2, 1:4, 1:8, and 1:16 and also 0:1 (labeled monomer alone) were added to each array and incubated for 4 to 5 h at RT in the dark under slow shaking. After incubation, each array was washed three times with block buffer and left to dry before scanning using a Typhoon Trio scanner (GE Life Sciences). Densitometry quantification of each spot was done using the ImageJ Protein Array Analyzer toolbox (Wayne Rasband, National Institutes of Health) (99).

### Peptide handling

Selected peptides were synthesized to 75% purity by GenScript Company. The lyophilized peptides were weighed out, dissolved in MilliQ water, and the concentration of stock solutions was determined using calculated ε_280_ values. For peptides lacking Trp and tyrosine (Tyr), concentrations were estimated on weight, based on the ratio between the concentrations of Trp/Tyr-containing peptides estimated from weight *versus* absorption at 280 nm. The stock solutions were stored at −80 °C until further use.

### Trp fluorescence anisotropy

Fluorescence anisotropy of Trp-containing peptides (0.1 mg/ml) in the presence of αSOs (0.2 mg/ml) was recorded in a 10-mm quartz cell on a LS-55 Luminescence fluorimeter (PerkinElmer Instruments) over 5 min at 25 °C using both parallel and perpendicular polarizers. Excitation at 295 nm and emission spectra obtained over 340 to 350 nm were determined using 10-nm slit widths for both excitation and emission wavelengths and an integration time of 5 s.

### Plate reader seeded and unseeded fibrillation assays

About 150 μl of 70 μM (1 mg/ml) α-syn, 40 μM ThT, and varying amounts of peptides (0, 0.06, 0.125, 0. 25, 0.375, and 0.5 mg/ml) were transferred in triplicates to a black COSTAR 96-well clear bottom plate (Nunc, Thermo Fisher Scientific), and including a 3 mm diameter glass bead (Glaswarenfabrik Karl Hecht GmbH & Co KG) per well to increase reproducibility (100). The plate was sealed with Crystal Clear sealing tape (Hampton Research) and incubated at 37 °C in a CLARIOstar Plus (BMG LABTECH) fluorescence plate reader. Fibrillation was carried out for ∼48 h with 300 rpm double orbital shaking between the readings for 372 s (excitation/emission: 448∕485 nm). Seeding experiments were performed using the same setting as for the fibrillation assay as described (39). Briefly, the experiments were done in triplicates in the presence of 0.05 mg/ml of α-syn fibril seeds (5% seeding) and 1 mg/ml of peptides in a solution of 1 mg/ml α-syn in 96-well plates.

### SDS-PAGE gel analysis of α-syn–peptide samples

To determine the amount of soluble α-syn, fibrillated samples were centrifuged (13,000 rpm, 15 min) and the supernatants were loaded on Bis–Tris 15% acrylamide gel with prestained protein ladder (Thermo Scientific) and run in a Bis–Tris running buffer at 150 V for 70 min, stained for 20 min in Coomassie brilliant blue (1.2 mM Coomassie brilliant blue, 5% ethanol, and 7% acetic acid), and destained overnight in a destaining solution (5% ethanol and 5% acetic acid). Gel bands were quantified using ImageJ.

### Far-UV CD of fibrillated α-syn–peptide samples

To remove free α-syn and peptides, fibrillated samples were centrifuged (13,000 rpm, 15 min). This leaves monomeric α-syn plus unbound and free peptides in the supernatant. The supernatant was discarded, and the pellet was washed two times in the same volume of PBS buffer, pH 7.4, to ensure complete removal of monomeric α-syn and free peptides. Far-UV CD spectra were recorded at 25 °C on a Chirascan CD Spectrophotometer (Applied Photophysics) using a 0.1 mm quartz cuvette in the wavelength range of 200 to 260 nm with 1 nm bandwidth and three accumulations. The buffer spectrum was subtracted, and the results were expressed as mean residue ellipticity [θ] (deg cm^2^ dmol^−1^).

### ATR–FTIR of fibrillated α-syn–peptide samples

The samples prepared in the CD section were also used to measure FTIR spectra on a Tensor 27 FTIR spectrophotometer (Bruker). About 2 μl α-syn fibrils were dried on the ATR crystal with a gentle stream of nitrogen gas. Spectra were accumulations of 68 scans in the 4000 to 1000 cm^−1^ range. Baseline correlation, second derivative, and atmospheric compensation were performed with OPUS 5.5 software (Bruker, Germany). Signals in the amide I region (∼1550–1700 cm^−1^) were smoothed and deconvoluted with PeakFit, version 4.12 software (ِSystat Software Inc) using Lorentzian fitting.

### TEM

Five microliters of each fibril-containing solution were applied to 400-mesh carbon-coated and glow-discharged nickel grids for 30 s, and blotted with filter paper to remove excess sample. The grids were washed with one drop of double distilled water and then stained with 3 μl of 1% uranyl formate for 15 s. Excess stain was removed by blotting paper This step was repeated two more times. Finally, the grids were left to air dry and viewed on a Tecnai G2 Spirit (FEI Company) operating at 60 kV, and images were taken using a TemCam F416 camera (TVIPS).

### Preparation of calcein-filled LUVs

Calcein-filled vesicles of DOPG were prepared as described before (38). Briefly, DOPG was dissolved in PBS to 10 mg/ml instead of 5 mg/ml, in the presence of calcein at self-quenching concentrations (70 mM) and subjected to 10 freeze–thaw cycles between liquid nitrogen and a 50 °C water bath. After 21 times extrusion through a 100 nm filter, free calcein was separated from vesicles with a PD-10 desalting column (GE Healthcare). Vesicles were stored at 4 °C and used for calcein release assays within 24 h.

### Calcein release assays

Calcein release was measured by mixing peptides and/or αSOs at a final concentration of 10^−6^ to 10^−1^ mg/ml and 5.2 μg/ml, respectively, in 148 μl with 2 μl of calcein vesicles, and were then loaded onto a 96-well plate (Nunc, Thermo Fischer Scientific) in triplicate. The plate was sealed with Crystal clear sealing tape (Hampton Research), and fluorescence was measured (excitation: 485 nm and emission: 520 nm) for 1 h at 37 °C every 2 min with a 2 s autoshake before each reading on a fluorescence plate reader (Tecan). Finally, 2 μl Triton X-100 (0.2% [v/v]) was added to lyse vesicles, and the maximal fluorescence signal was measured for 20 min under the same reading condition. Background fluorescence was subtracted, and the average signal of calcein release before addition of Triton X-100 (F), after treatment with Triton X-100 (F_t_) and for the buffer control (F_0_) were determined, and calcein release percentage was calculated as follow (Equation 1):

(1) $$\text{Calcein}\;\text{Release}\%\;\left(\text{CR}\%\right)=\frac{\left(F-F_{0}\right)}{\left(F_{t}-F_{0}\right)}$$

### Peptide labeling with fluidiphore rapid amine 503

Peptides were labeled with fluidiphore rapid amine 503 (Fluidic Analytics Limited), an amine-reactive fluorescent label whose absorbance shifts from 612 nm (free) to 503 nm when conjugated. About 1 mg dry fluidiphore dye was dissolved in 100 μl acetonitrile and stored at −20 °C. The peptide stock solutions were diluted into labeling buffer (0.1 M NaHCO_3_, pH 8.3) to a final concentration of 1 mg/ml (100 μl) and mixed with 3 μl of dye solution (final dye concentration of 0.3 μg/ml) and incubated for 30 min at RT. The solutions were centrifuged at 14,000*g* for 15 min to remove unreacted dye that precipitates out.

### Binding affinity measurement

Fluidity One-W (Fluidic Analytics Limited) (101) was used to measure the binding affinity and *R*_*h*_ of peptides bound to α-syn monomers and oligomers. We determined the lowest concentration of labeled peptides that could be detected by the instrument to be 60 μM. Subsequently, samples with 60 μM of labeled peptides and different concentrations of α-syn monomers and oligomers (0–400 μM and 0–84 μM, respectively) were made using MilliQ water and incubated 15 min in the dark before measurement. Data were analyzed by GraphPad Prism 8 (GraphPad Software, Inc) software using one-site (Equation 2) and multisite (Equation 3) fitting models for α-syn monomers and oligomers, respectively (66):

(2) $$Y=\frac{B_{\max}\times X}{\left(K_{d}+X\right)}+\text{NS}\times X+\text{Background}$$

(3) $$Y=\frac{B_{\max}\times X^{h}}{\left({K_{d}}^{h}\times X^{h}\right)}$$

where X is peptide concentration, Y is fraction bound, B_max_ is maximal fraction bound, *K*_*d*_ is the peptide equilibrium dissociation constant, and h is the Hill coefficient. We also include the term NS (slope) to take into account nonspecific binding and a background term.

### Evaluation of cell viability: measurement of cell viability by MTT assay

We used the MTT assay to measure cell viability after 24 h treatment with αSOs in the presence of peptides. SH-SY5Y cells were seeded in 96-well plates at a density of 5 × 10^4^ cell/ml in Dulbecco's modified Eagle's medium supplemented with 10% FBS, 100 unit/ml penicillin, and 100 mg/ml streptomycin. The cells were incubated in a humidified atmosphere incubator with 5% CO_2_ and 95% humidity at 37 °C for 24 h. The culture medium was then replaced with fresh medium containing 10 μM αSOs with peptides and incubated for another 24 h. Then the cell medium was replaced with a fresh medium containing 20% MTT (5 mg/ml), and the cells were incubated for an additional 4 h at 37 °C. Then 100 μl of dimethyl sulfoxide was added to dissolve formazan crystals by incubating for 30 min on a shaker at RT. Consequently, absorbance was measured by using a plate reader at 570 nm.

### Statistical methods

Each experiment was performed twice in triplicate. Data were expressed as mean ± SD, and the statistical analysis was done using GraphPad Prism 8.0 software). The control and treated samples were compared using unpaired *t* tests. A *p* value < 0.05 was considered as statistically significant.

## Data availability

The data that support the findings of this study are available from the corresponding author upon reasonable request.

## Supporting information

This article contains supporting information (supplemental Figs. S1–S9, Tables S1 and S2).

## Conflict of interest

The authors declare that they have no conflicts of interest with the contents of this article.
