# Supplementary material for: A penetratin-derived peptide reduces the membrane permeabilization and cell toxicity of α-synuclein oligomers
Source: J Biol Chem. 2022 Nov 10;298(12):102688. doi: 10.1016/j.jbc.2022.102688 (PMC9791135; doi:10.1016/j.jbc.2022.102688)
Supplement: Supporting information [file mmc1.docx]

**Supporting Information**

A penetratin-derived peptide reduces the membrane permeabilization and cell toxicity of α-synuclein oligomers

Mitra Pirhagh, Signe Andrea Frank, Parvez Alam, Janni Nielsen, Vita Sereikaite, Arpit Gupta, Kristian Strømgaard, Maria Andreasen, Deepak Sharma, Ali Akbar Saboury*, Daniel Erik Otzen*

**Table S1**. Celluspots^TM^ peptide array, Peptide sequences

| Spot number | Peptide name |
| --- | --- |
| 1-127 | 14-mers of alpha-synuclein sequence |
| 128-185 | Sequenced bacteriophage peptides |
| 186-201 | Alanine scanning of penetratin (RQIKIWFQNRRMKWKK) |
| 202-211 | C-terminal truncation of penetratin |
| 212-221 | N-terminal truncation of penetratin |
| 222-234 | Modulating hydrophobicity of penetratin |
| 235-254 | Scrambled version of penetratin |
| 255-268 | Alanine scanning of KLA peptide (KLAKLAKKLAKLAK) |
| 269-279 | Modulating hydrophobicity of KLA peptide |
| 280 | N- and C-terminal truncation |
| 281-284 | combination based on sequence logo |
| 285-299 | Modulating hydrophobicity of peptide SACNHHHHLHCGG |
| 300-329 | Scrambled version of peptide SACNHHHHLHCGG |
| 330-354 | Combination based on sequence logo from peptide SACKTPSWRFCGG |
| 355-374 | Scrambled version of peptide SACKTPSWRFCGG |
| 375-379 | Modifications of peptide SACNHHHHLHCGG |
| 380 | Negative control |
| 381 | N1C1-terminal truncation of penetratin |
| 382 | N2C2-terminal truncation of penetratin |
| 383 | N3C3-terminal truncation of penetratin |
| 384 | N4C4-terminal truncation of penetratin |

**Table S2**. Detailed secondary structure of peptides.

| Peptides | Secondary structure | | | | | |
| --- | --- | --- | --- | --- | --- | --- |
|  | Coil | Random | α-Helix | Parallel β-sheet | Anti-parallel β-sheet | Cross β-sheet |
| p5 | ** | * |  | * | ** | ** |
| p12 | * | * | * | * | ** | ** |
| p162 | * | * |  | * | ** | ** |
| p168 | ** | ** |  |  | * | ** |
| p216 | * |  |  | * |  |  |
| p222 | * |  |  |  | * | ** |
| p194 | * | * |  |  | ** | ** |
| p235 | * |  | ** |  |  | * |
| p249 | * | * |  |  | ** | ** |

**
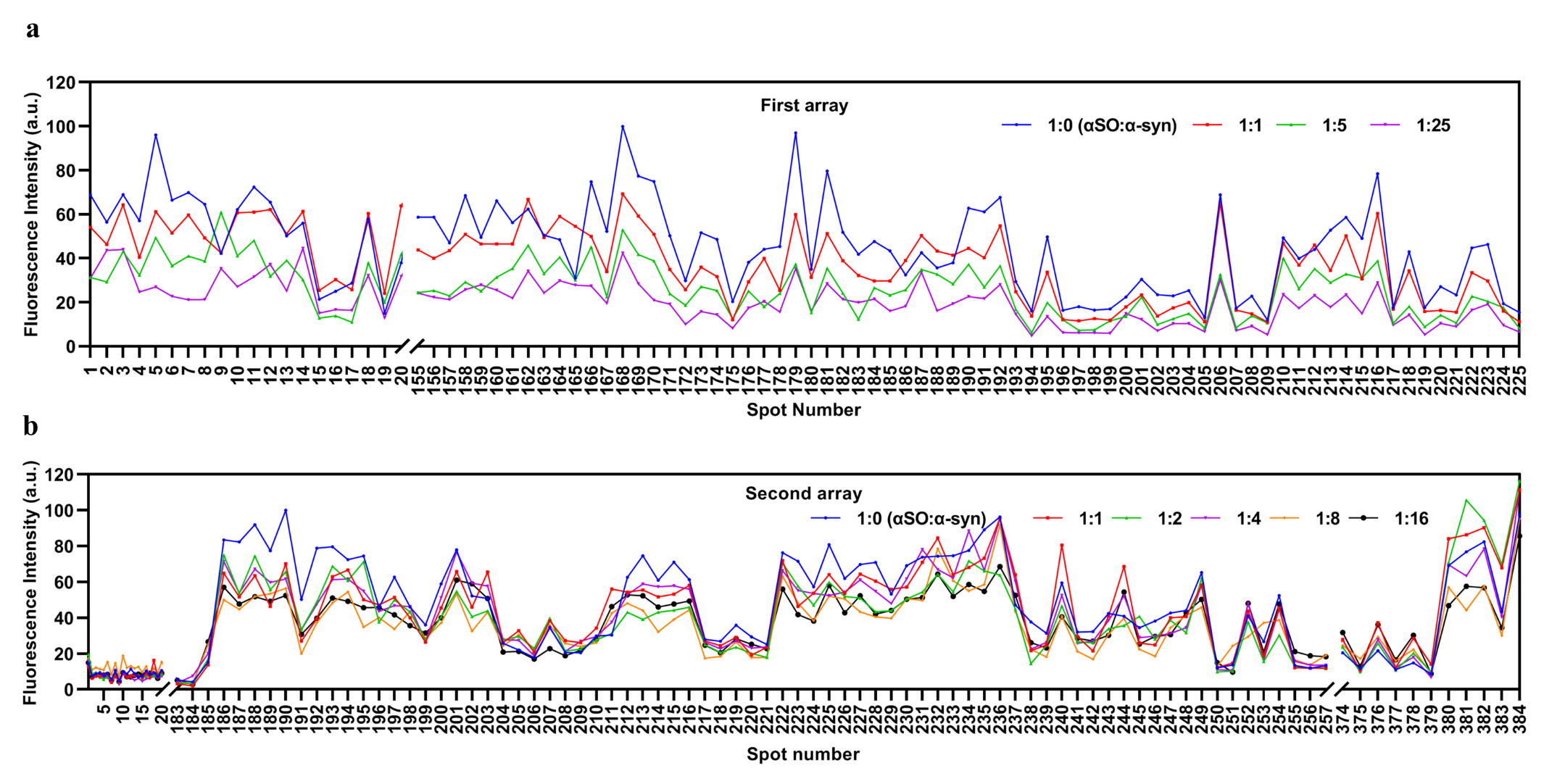
Figure S1**. Fluorescence intensity of labelled αSOs after incubation with 384 peptides on two identical peptide arrays. Labeled αSOs was incubated with unlabeled α-syn at the indicated oligomer: monomer mass ratios to ascertain how well monomers could displace αSOs from the different peptides.


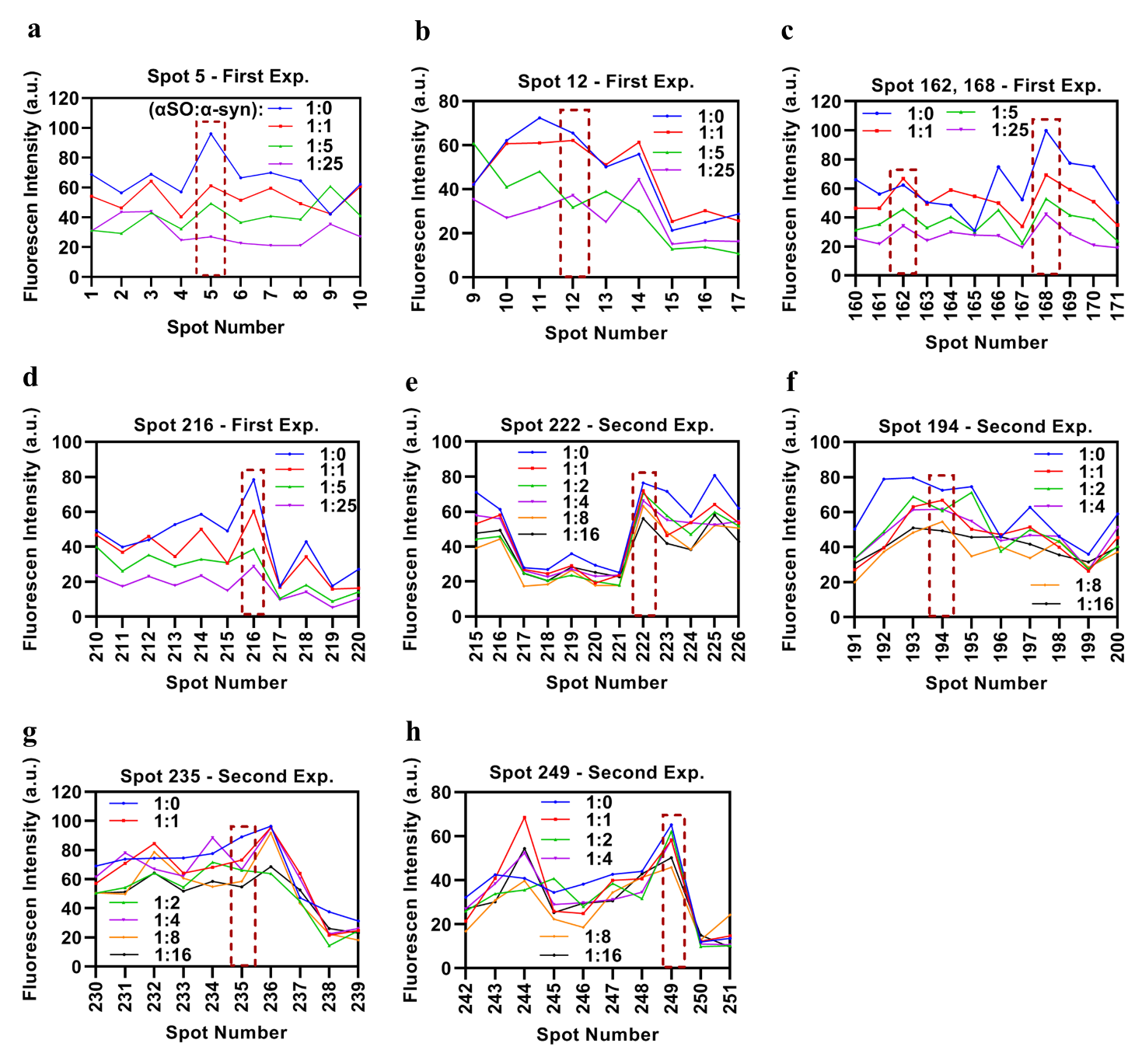


**Figure S2**. Zoom of Fig. S1 in the region around the 9 selected peptides.

**
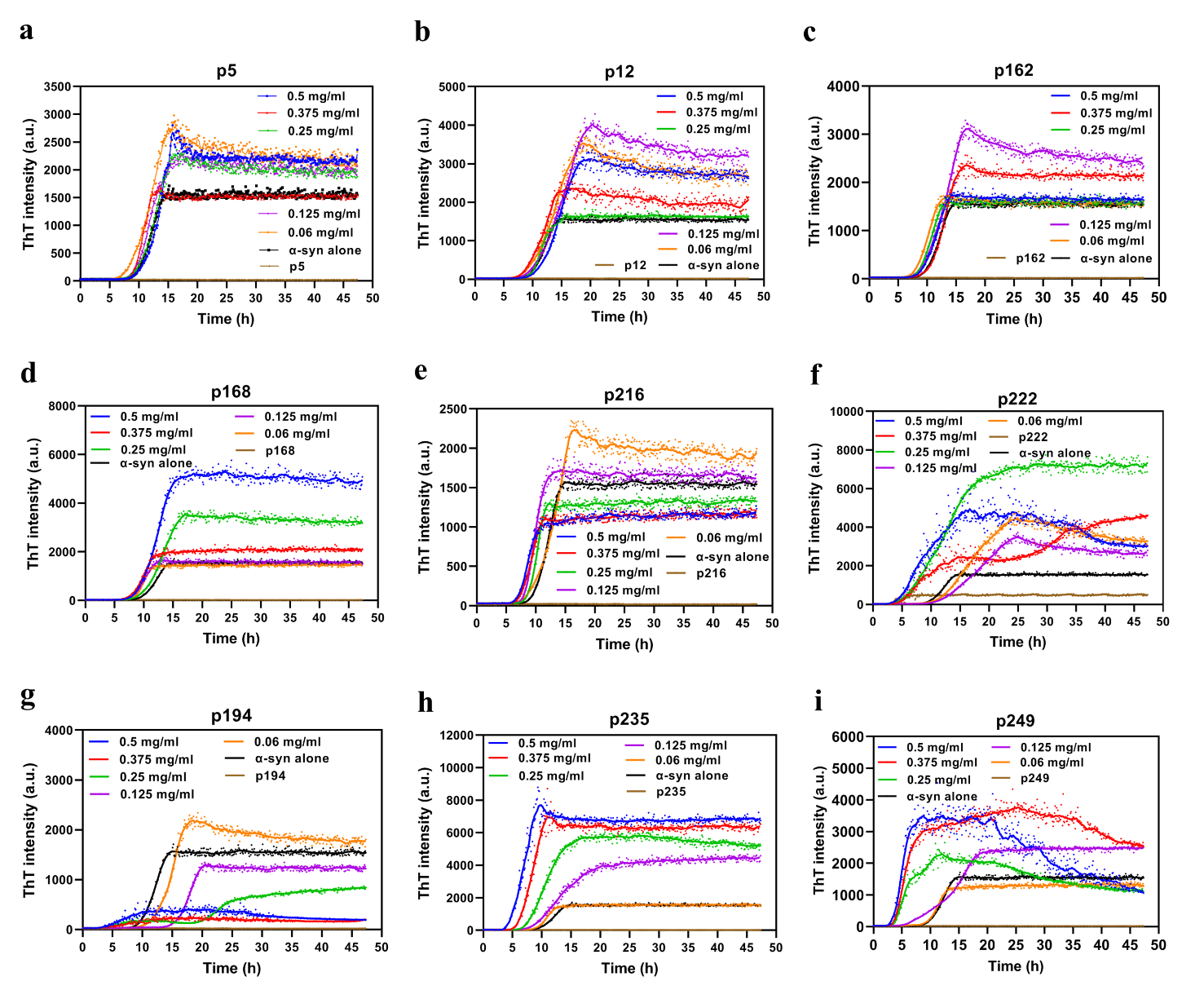
Figure S3**. The effect of the 9 peptides on α-syn fibrillation monitored by ThT fluorescence. 1 mg/mL α-syn was incubated at 37°C with shaking in the presence of 0.06-0.5 mg/mL peptide. Peptide controls were incubated in 1 mg/mL.

**
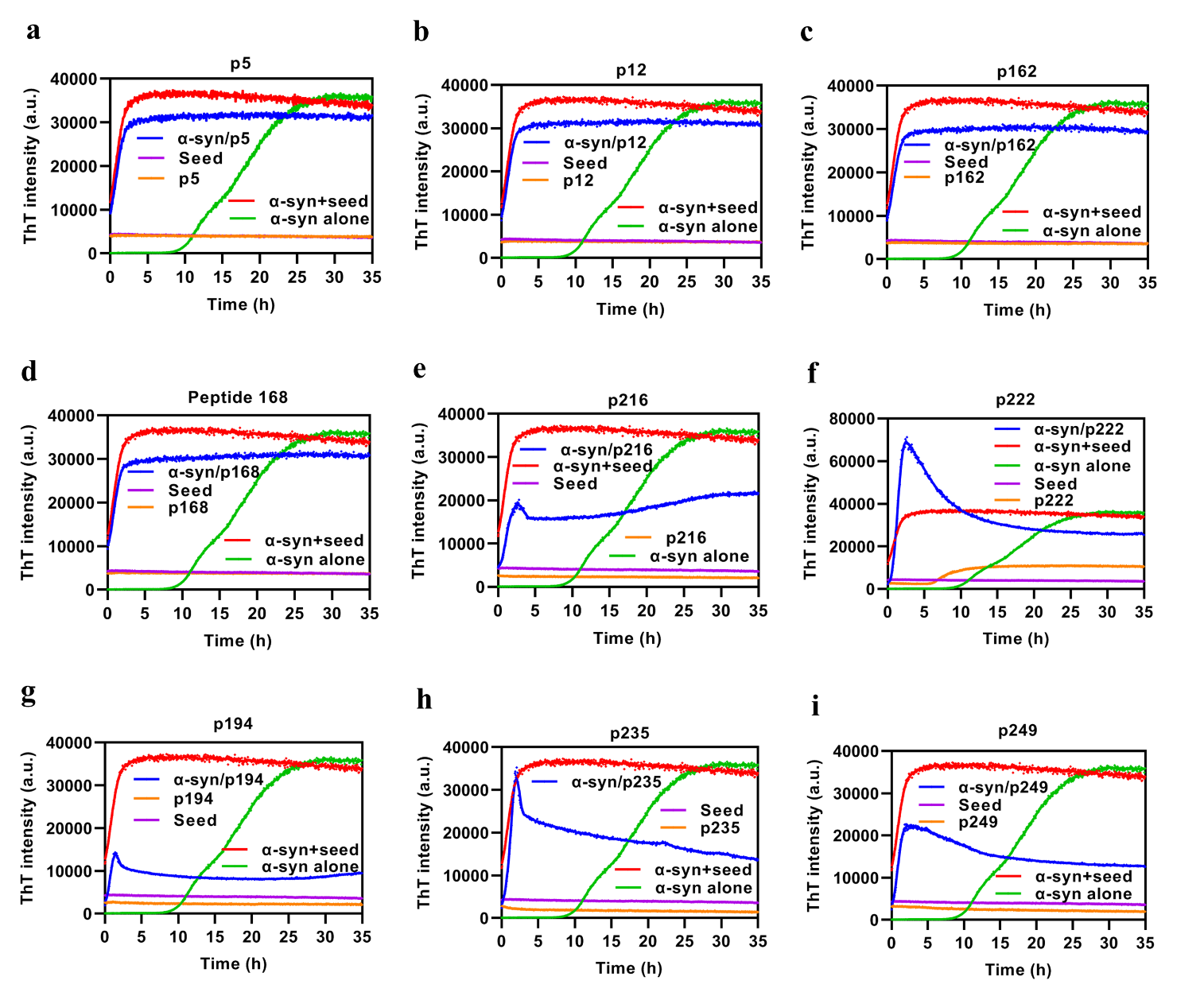
Figure S4**. The effect of the 9 peptides on α-syn secondary nucleation and elongation. (a-i) ThT fluorescence assays of samples with 1 mg/mL α-syn incubated at 37°C with shaking in the presence of 5% α-syn seeds and 1 mg/mL of the indicated peptides.

**
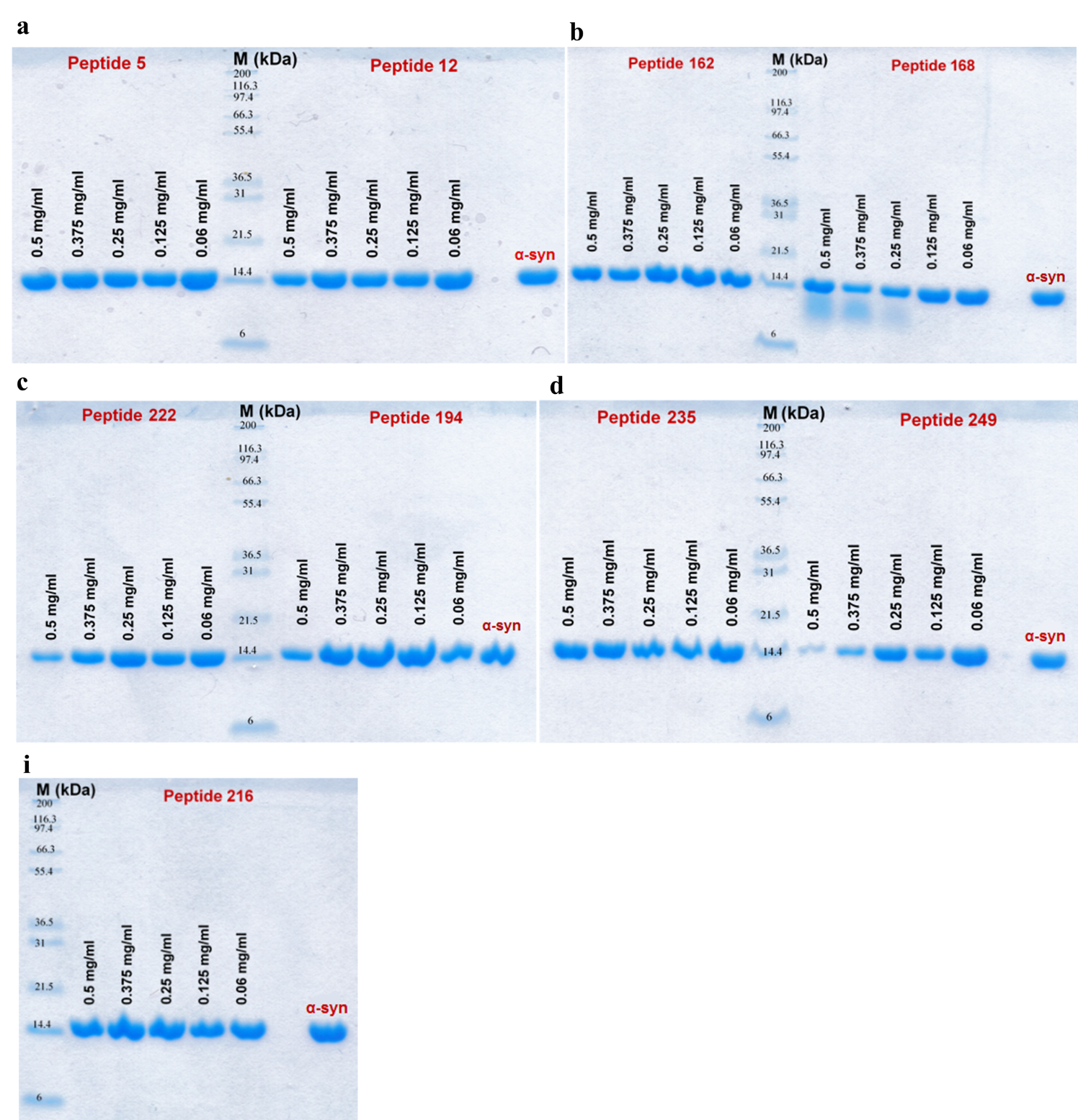
Figure S5**. SDS-PAGE for supernatants of all the samples to compare the amount of α-syn monomers left in the supernatants (not fibrillated) after the end of incubation. “α-syn” refers to α-syn fibrillated without any peptides present (lane 1 from right).

**
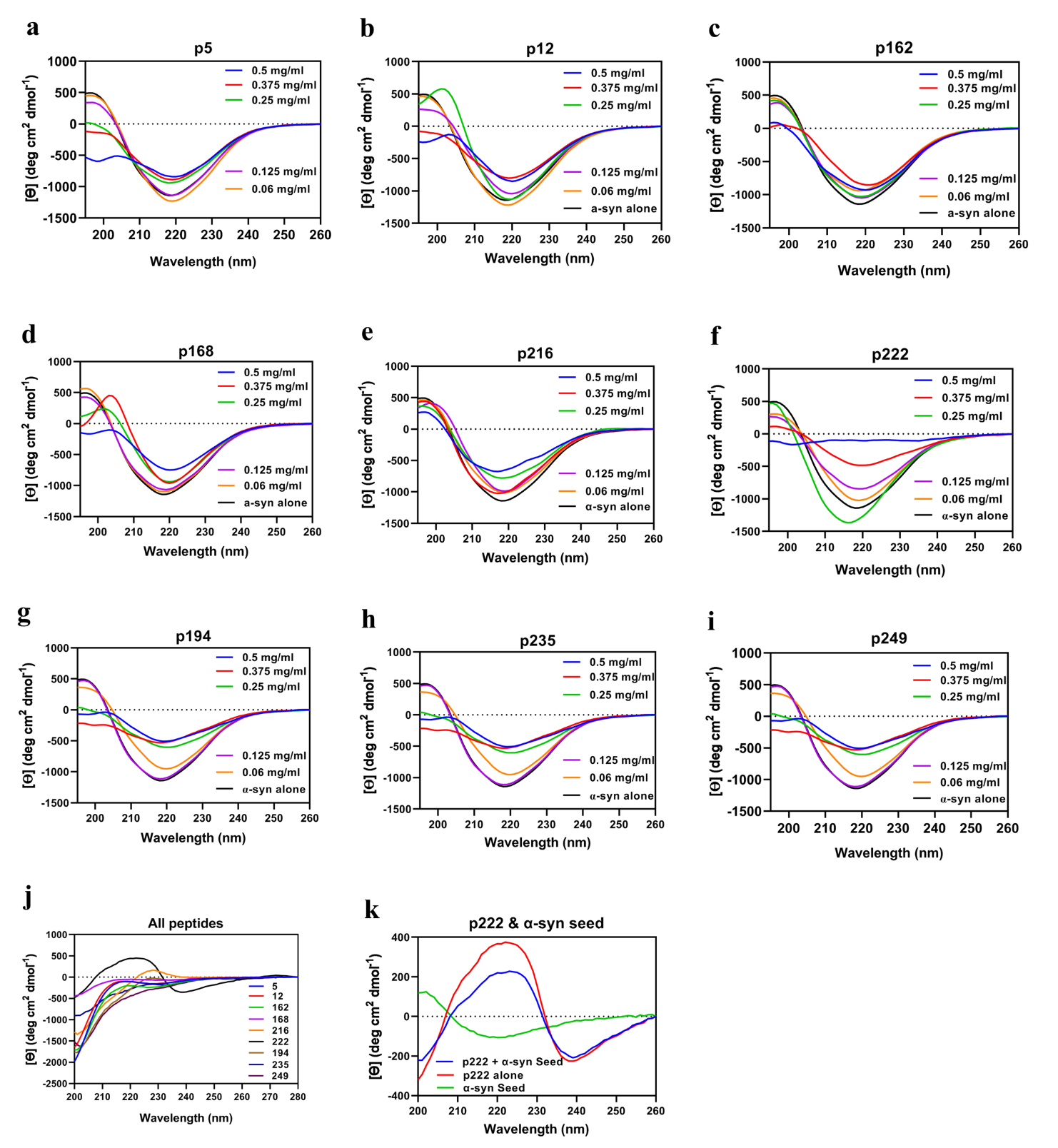
Figure S6**. Far-UV CD spectra of α-syn samples after 48 h of incubation of 1 mg/ml α-syn in the presence of 0-0.5 mg/ml peptide (panels a-i). In addition, we include (j) far-UV CD spectra of peptides alone after incubation. (k) Far-UV CD spectra of peptide 222 aggregated after incubation in the presence and absence of α-syn seeds.

**
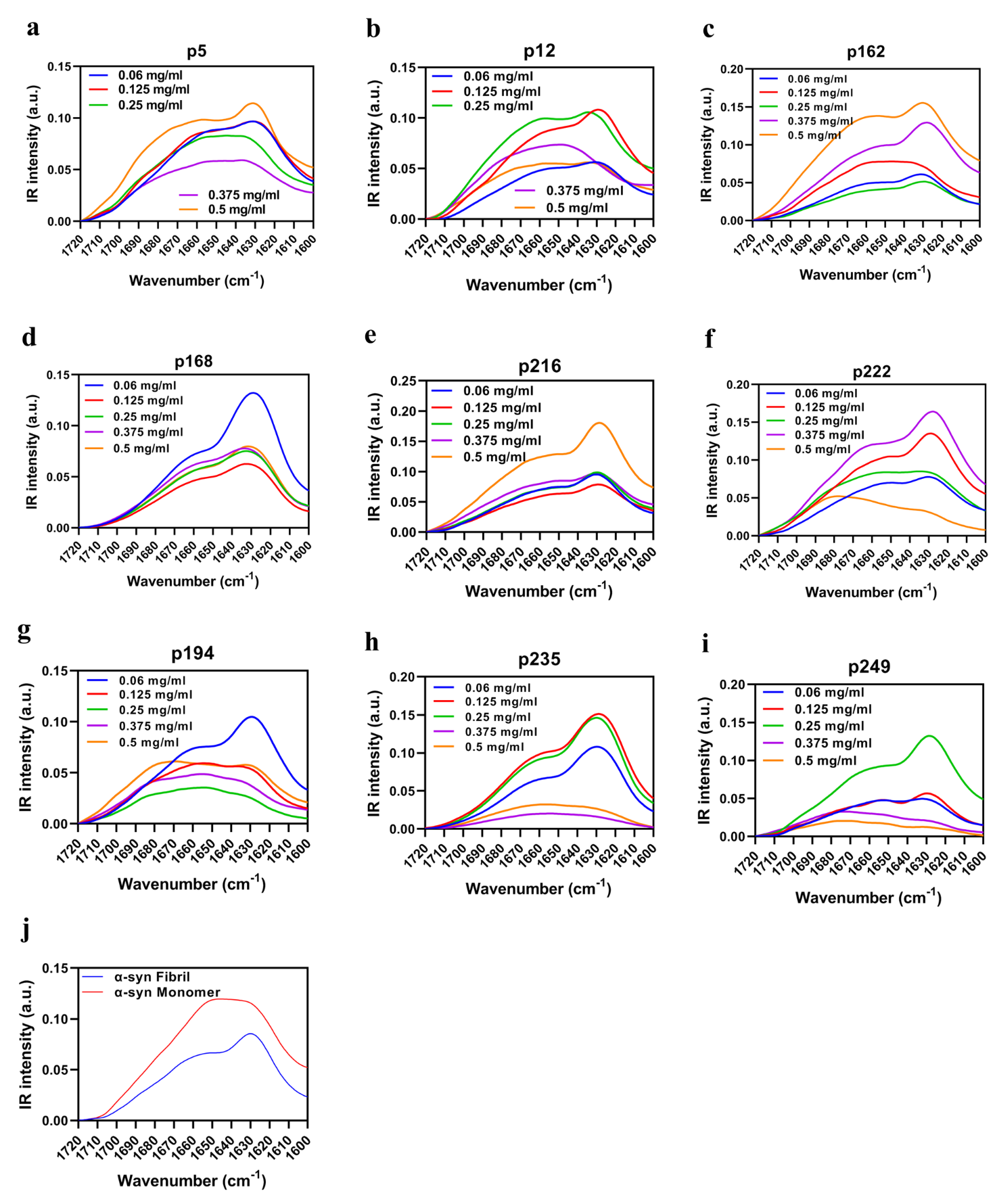
Figure S7**. ATR-FTIR spectra for of samples after 48 h incubation. (a-i) α-syn in the presence of different concentrations of peptides. (j) α-syn monomer and fibril alone.


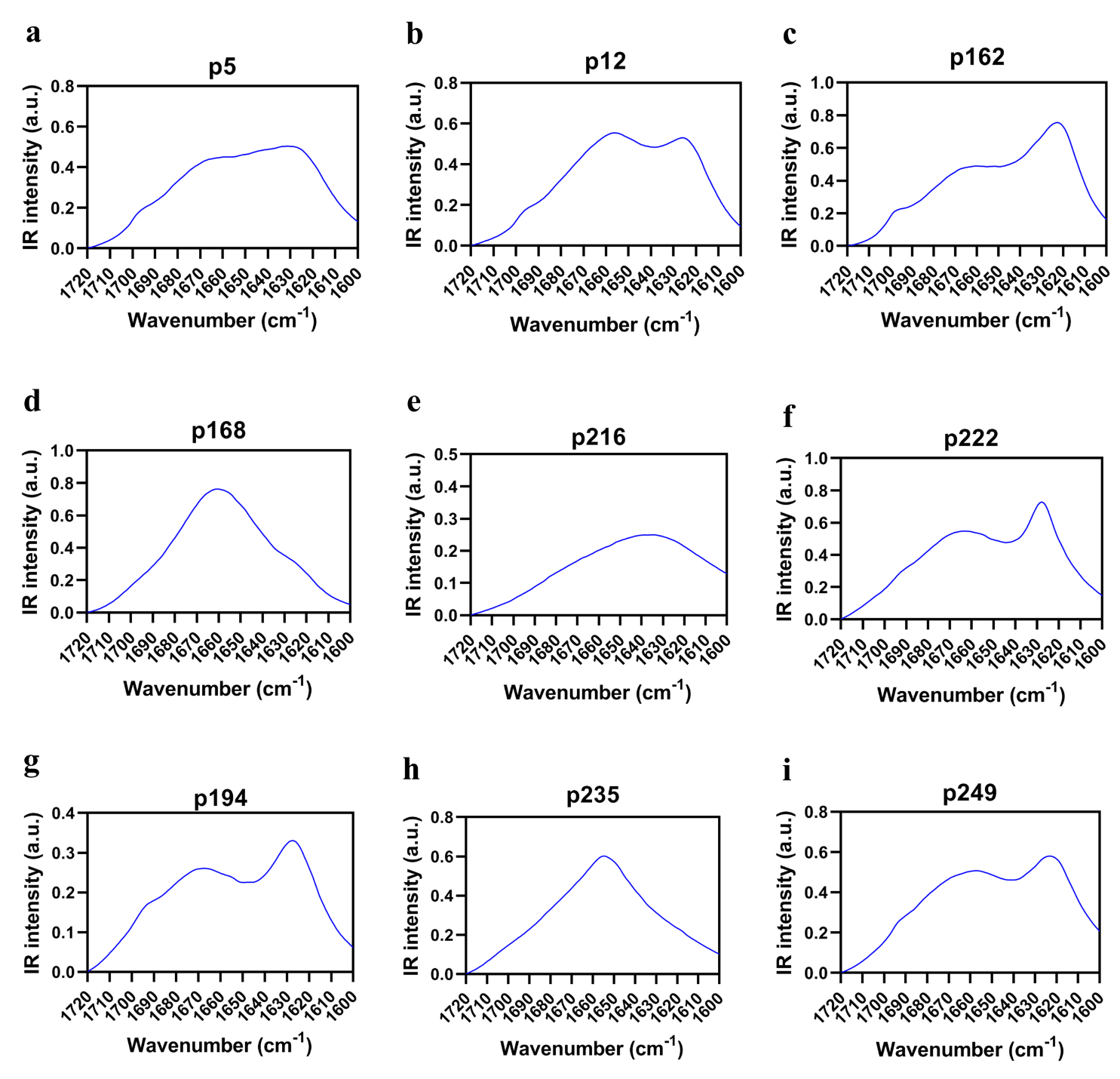


**Figure S8**. ATR-FTIR spectra of peptides alone.

**
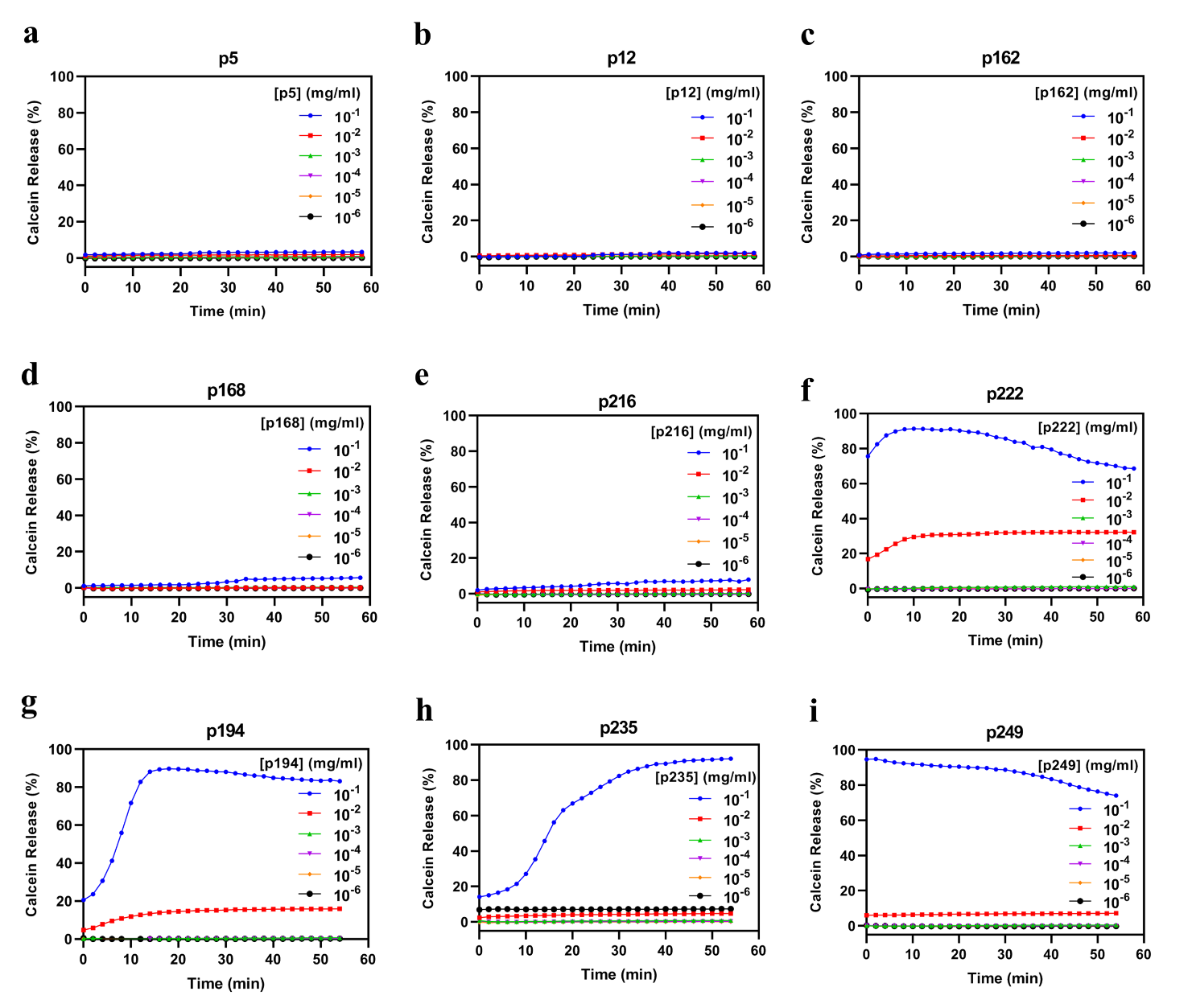
Figure S9**. Time profiles of calcein release from calcein-loaded DOPG vesicles in the presence of different peptides concentrations in the absence of αSO.

**
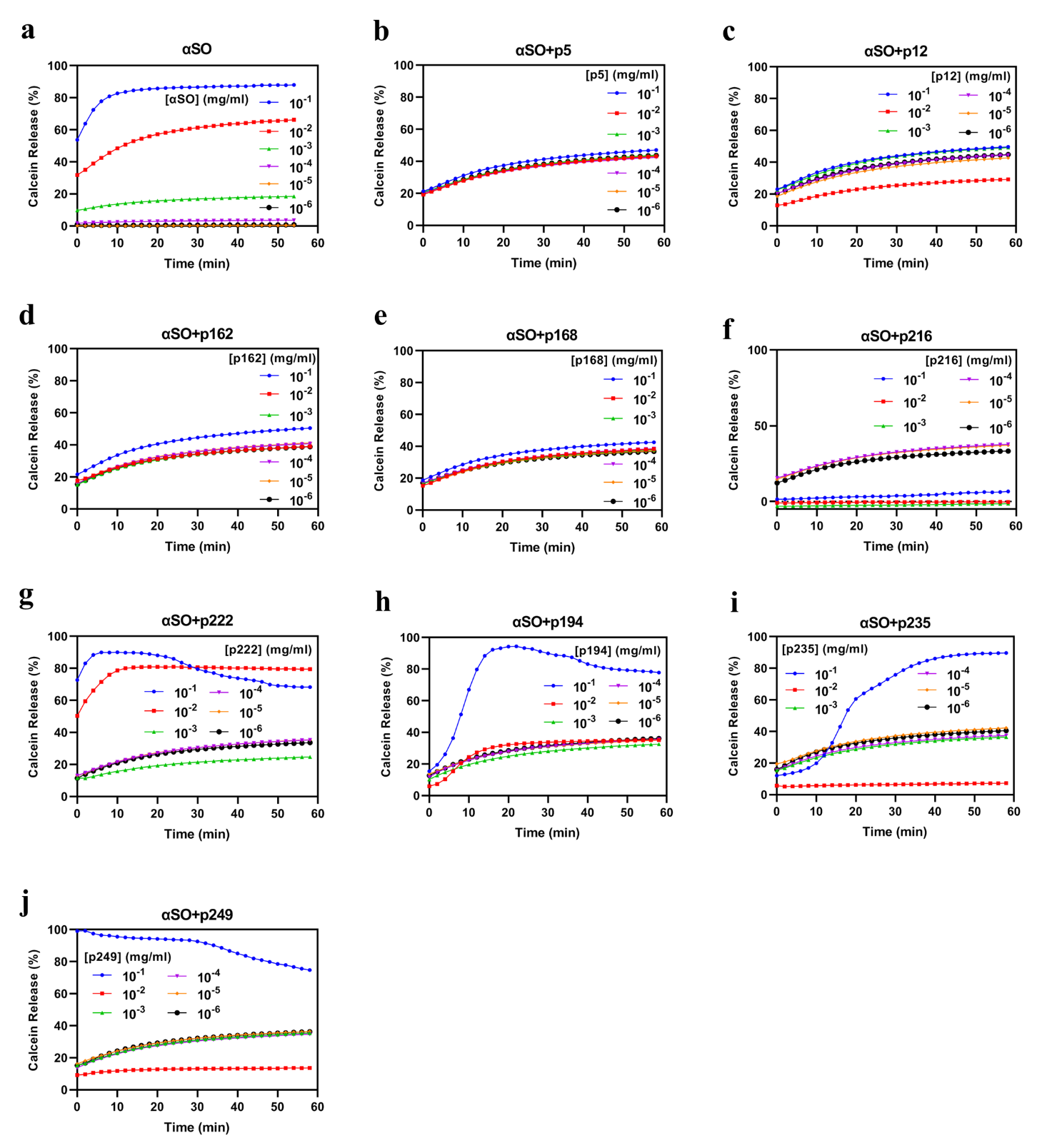
Figure S10.** Time profiles of calcein release from calcein-loaded DOPG vesicles in the presence of (a) αSO and (b-j) αSO/peptides.


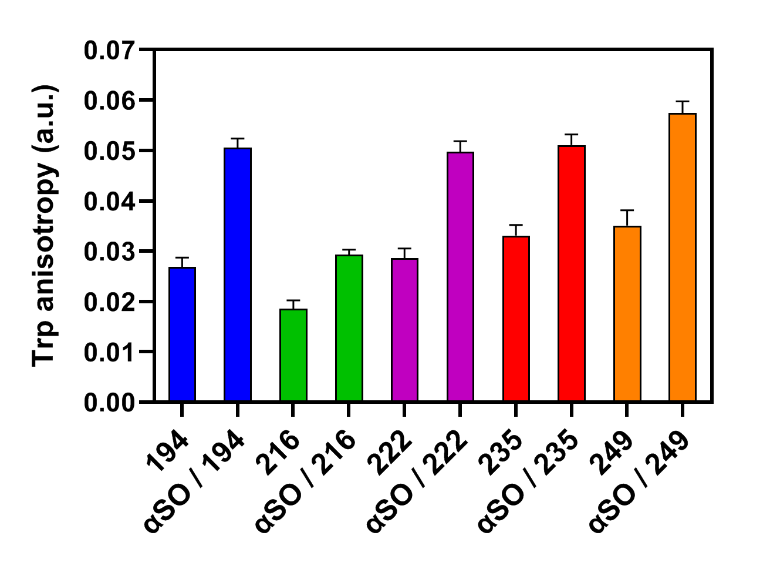


**Figure S11**. Fluorescence Trp anisotropy of Trp containing peptides alone (0.1 mg/ml) and in the presence of αSOs (0.2 mg/ml).
